# New diagnostic technique to evaluate hepatic steatosis using the attenuation coefficient on ultrasound B mode

**DOI:** 10.1371/journal.pone.0221548

**Published:** 2019-08-27

**Authors:** Yohei Koizumi, Masashi Hirooka, Nobuharu Tamaki, Norihisa Yada, Osamu Nakashima, Namiki Izumi, Masatoshi Kudo, Yoichi Hiasa

**Affiliations:** 1 Departments of Gastroenterology and Metabology, Ehime University Graduate School of Medicine, Toon City, Ehime, Japan; 2 Department of Gastroenterology and Hepatology, Musashino Red Cross Hospital, Tokyo, Japan; 3 Department of Gastroenterology and Hepatology, Faculty of Medicine, Kindai University, Osaka, Japan; 4 Pathology Division, Kurume University Hospital, Fukuoka, Japan; Policlinico Universitario Campus Bio-Medico, ITALY

## Abstract

**Purpose:**

We have developed a diagnostic technique to evaluate hepatic steatosis using the attenuation coefficient (ATT) in ultrasound B mode imaging. A controlled attenuation parameter (CAP) by vibration-controlled transient elastography (VCTE) has also been used to evaluate hepatic steatosis. As that method uses ultrasound A mode, visualizing the liver in real time is difficult. We designed this clinical study to evaluate the diagnostic advantage of our technique using ATT compared to CAP.

**Materials and methods:**

The study group included 94 patients with chronic liver disease who had undergone both ATT and CAP assessment at the time of liver biopsy. The M-probe and XL-probe were used for CAP measurement. Data for ATT and CAP were compared as a function of the steatosis grade.

**Results:**

The area under the receiver operating characteristic curve (AUC-ROCs) for ATT and PAC as a function of the steatosis grade were as follows: grade 1, 0.74 and 0.81; grade 2, 0.80 and 0.85; and grade 3, 0.96 and 0.98, respectively.

**Conclusion:**

The accuracy of steatosis grade diagnosis using ATT was the same as that using CAP, with no significant differences and with the added advantage of B mode ultrasound being more convenient and rapid, compared to A mode ultrasound, particularly for patients with subcutaneous fat thickness ≥2 cm.

## Introduction

With the increase in the obese population, liver steatosis is one of the most common chronic liver diseases (CLDs) [1–3]. In addition, hepatic steatosis is also caused by alcohol consumption [4]. As hepatic steatohepatitis may progress to end-stage liver diseases, including cirrhosis and hepatocellular carcinoma [4–6], early and accurate diagnosis of hepatic steatosis is important to inform for proper management of patients with CLD [5,6].

In current clinical practice, the most used method for steatosis quantification is ultrasound B mode examination with identification of “bright liver,” “deep beam attenuation,” “vessel blurring,” and “liver to kidney contrast” and calculation of the Hamaguchi score [7]. The development of non-invasive techniques, such as vibration-controlled transient elastography (VCTE), for the diagnosis of liver fibrosis has been reported in a number of recent studies [8–12]. Use of the controlled attenuation parameter (CAP) as a non-invasive assessment of hepatic steatosis has been proposed, with several recent studies having shown a significant correlation between CAP and the steatosis grade in patients with different pathogenesis of CLD [13–17]. However, VCTE is measured from a single shear wave based on ultrasound A mode and, thus, the section of the liver being measured cannot be observed in real time. Moreover, VCTE cannot be performed postoperatively (such as after right hepatic lobectomy) or in the presence of ascites. Furthermore, as real time visualization during VCTE measurement is not possible, it may not be clear if the region from which measurements are obtained includes structures other than liver parenchyma, such as vessels within the liver [8]. In patients with severe obesity, use of only one probe does not allow complete measurement of the accumulation of steatosis, with a change to an XL-probe being necessary. Overall, it is clear that better ultrasound-based methods of assessment of liver steatosis are needed.

One potential solution to the problems of CAP is a new diagnostic method using the attenuation coefficient (ATT) measured on ultrasound B mode imaging [18]. Ultrasound B mode uses multiple ultrasound wave with different frequency components for measurement. Thus, ATT estimates hepatic steatosis from differences in attenuation of the received signals. Since ATT is based on B mode, real time visualization of the target area for measurement is available [18]. Furthermore, ATT measurements are obtained at a depth of 40–100 mm, avoiding the influence of subcutaneous fat thickness, avoiding the need for a change in probe for obese patients. Given these factors, we considered ATT to be potentially advantageous in terms of reproducibility and success rate for assessing hepatic steatosis. The objectives of this prospective study were, therefore, to compare the diagnostic accuracies of CAP and ATT for the assessment of hepatic steatosis among patients with CLD.

## Materials and methods

### Patients

**Fig 1:**
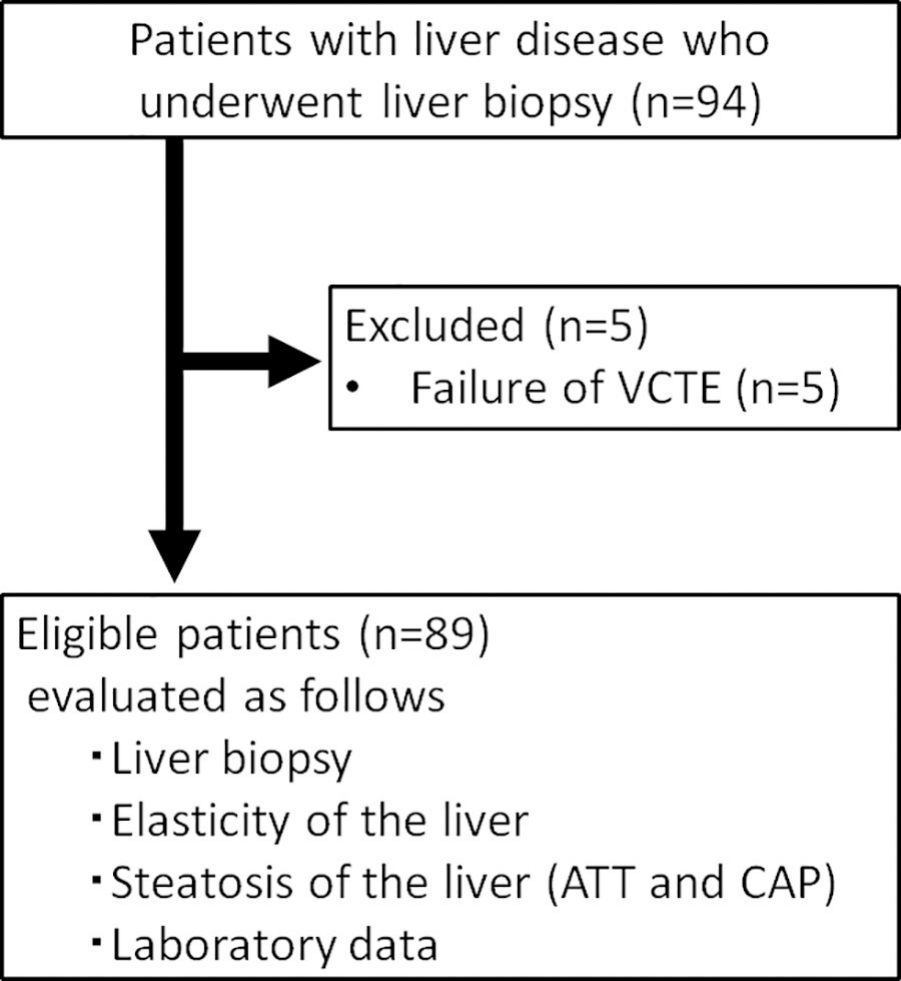
Flow chart of the study participants. VCTE, vibration-controlled transient elastography; ATT, attenuation coefficient; CAP, controlled attenuation parameter.

Written informed consent was obtained from all study participants before enrollment, and all study protocols were approved by our institutional ethics committee (Certified Review Board Ehime University, permit number: 1509016). The analysis was based on a component of multicenter research data [18]. Ninety-four patients with liver disease, who underwent liver biopsy between July 2015 and March 2017, were enrolled into our study. All patients underwent measurement using both CAP and ATT. The inclusion criterion was age ≥20 years. The exclusion criterion was failure of VCTE measurements (n = 5). We performed a prospective performance analysis of ATT and CAP for the diagnosis of steatosis according to the non-alcoholic fatty liver disease (NAFLD) activity score (NAS) using a receiver operating characteristic (ROC) analysis (Fig 1). The primary endpoint was evaluation of the diagnostic advantage of ATT compared to CAP, by correlating the index calculated using ATT measurement to the CAP measurement.

### Measurement of hepatic steatosis

**Fig 2:**
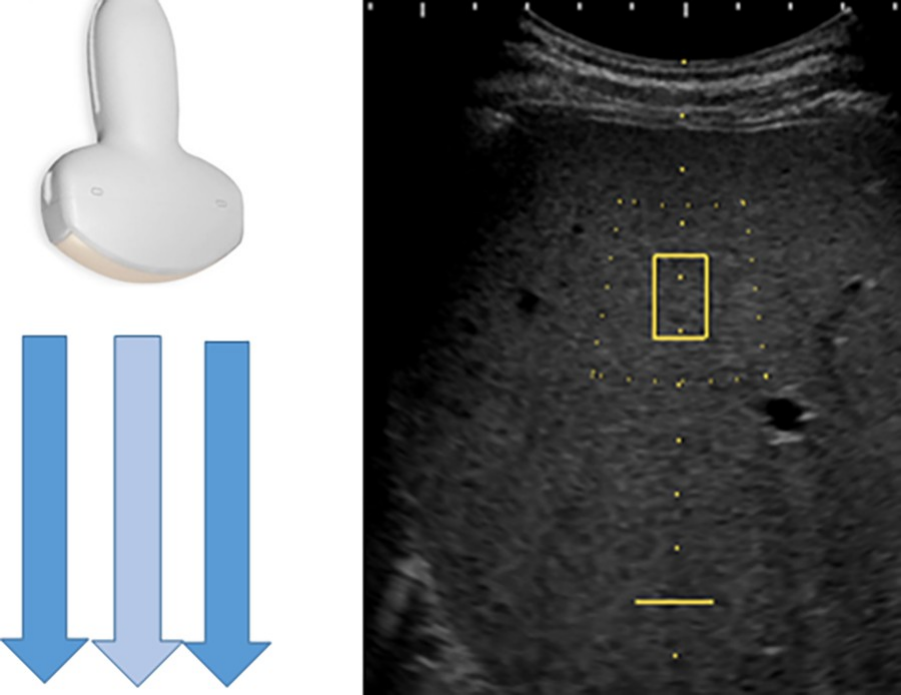
Diagnosis of fatty liver using attenuation in the B mode. Multiple ultrasound waves with different frequency components are used when measuring in the B mode. Fat volume estimation is performed according to the difference in the degree of attenuation of the received signal. Measurement is performed while observing the relevant part in real time.

After fasting overnight, ATT and CAP measures for hepatic steatosis were obtained on the same day as liver biopsy. Measures were obtained using the ultrasound system (HI VISION Ascendus; Hitachi, Tokyo, Japan), with the convex probe used for ATT (EUP-C715, 5–1 MHz, Hitachi) and CAP (Echosen Fibroscan 502, Paris, France). A total of 10 valid measurements were obtained for CAP in each patient. The median liver steatosis was calculated as previously described [13]. All measurements were recorded by two experienced gastroenterologists (Y.K. and M.H.), who had each conducted at least 350 liver stiffness evaluations prior to this study. ATT measurements were performed at the right intercostal space, without adding pressure from the probe (Fig 2). Measurements were obtained from regions of interest (ROIs) on the hepatic parenchyma, with hepatic steatosis then calculated. Ultrasound waves of different frequencies f_0_,f_1_ (f_0_<f_1_) were transmitted along the same beam line, and ATT was determined by calculating the slope of the received signal ratio (f_0_<f_1_). The median of five measurements was calculated as previously described [18]. In addition, measurement time and measurement success rate were recorded.

### Histological assessment of the liver

Liver biopsy was performed within 1 week of hospitalization, using a cutting needle, 1.6 mm in diameter and 150 mm in length [19]. As sampling error for identifying liver fibrosis may occur from such liver biopsies, samples less than 12 mm in length were excluded [20]. All liver biopsy samples were fixed in formalin and embedded in paraffin, and sections (4-μm-thick) were stained with hematoxylin-eosin and impregnated with silver [19]. Except for those classified as showing cirrhosis, liver biopsies comprising less than five portal tracts were excluded from histological analysis [18]. Fibrosis and the NAFLD activity score were evaluated by an experienced pathologist (O.N.), who was blinded to all patient characteristics. Liver steatosis was scored according to the NAFLD activity score, as follow: S0, <5%; S1, 5–33%; S2, 33–66%; and S3, >66% [21].

### Statistical analysis

Data were analyzed using Student’s *t* test for unpaired data and the chi-squared test and Fisher’s exact test, as appropriate for the data type. The correlation between ATT and CAP measurement and histological findings were analyzed using Spearman rank correlation analysis. Correlations between ATT and body mass index (BMI) were analyzed using Spearman rank correlation analysis. ROC curves were plotted, and the area under the curve (AUC-ROCs) were calculated using the trapezoidal rule. To maximize diagnostic accuracy, optimal cutoff values for liver stiffness were selected. Cutoffs obtained from ROC curves were then used to calculate sensitivity, specificity, and both positive and negative predictive values. Comparisons of CAP and ATT AUC-ROCs for the diagnosis of steatosis were performed using the DeLong test. The agreement between each observer’s ATT and CAP measurements was evaluated by calculating the kappa (κ) coefficient. The κ coefficient was interpreted as follows: κ <0.4, poor; 0.4 ≤ κ < 0.75, fair to good; and κ ≥0.75, excellent.

All data were analyzed using JMP (version 13, SAS Institute Japan, Tokyo, Japan). The DeLong test was analyzed using XLSTAT (Addinsoft Company, Paris, France).

## Results

### Patients

Among 94 patients who met the inclusion criteria, five patients were excluded because of unreliable VCTE measurements (no successful acquisitions with the M-probe).Thus, the analysis was based on the data of 89 patients. Characteristics of these patients at the time of biopsy are reported in Table 1. Of note, our study sample included 7 (7.9%) obese patients, with a BMI >30 kg/m^2^.

**Table 1:** Patient characteristics.

Characteristics	(n = 89)
Age (years)	65.0 ± 15.0
Sex (M:F)	89 (51:38)
BMI (kg/m^2^)	24.2 ± 3.9
AST (U/L)	101.5 ± 221.6
ALT (U/L)	141.6 ± 360.3
Serum albumin (g/dL)	3.92 ± 0.4
Platelet count (×10^4^/μL)	17.4 ± 7.1
Prothrombin time (%)	100.3 ± 17.2
Total bilirubin (mg/dL)	0.96 ± 1.0
Alkaline phosphatase (U/L)	357.1 ± 297.8
GGT (IU/L)	116.3 ± 174.1
Serum HA (μg/L)	84.3 ± 73.2
Etiology (HCV / HBV / NAFLD (NASH) / Alcohol / Other)	25 / 5 / 20 / 10 / 29
Histologic fibrosis stage	
F0	8
F1	42
F2	21
F3	10
F4	8
Histologic activity grade	
A0	6
A1	56
A2	26
A3	1
Histologic steatosis grade	
S0	63
S1	19
S2	5
S3	2

Data are expressed as mean ± standard deviation or n (%).

BMI, body mass index; AST, aspartate aminotransferase; ALT, alanine aminotransferase; GGT, gamma-glutamyl transferase; HA, hyaluronic acid; HCV, hepatitis C virus; HBV, hepatitis B virus; NAFLD, nonalcoholic fatty liver disease; NASH, nonalcoholic steatohepatitis

### Liver steatosis as assessed by ATT and CAP

**Fig 3:**
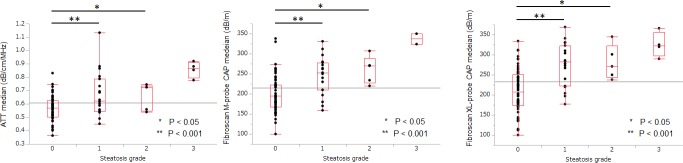
ATT and CAP values for each grade of steatosis. Graph shows the ATT value (A), CAP M-probe value (B) and CAP XL-probe value (C) for each steatosis grade. Vertical axis is a logarithmic scale. Tops and bottoms of the boxes = 1^st^ and 3^rd^ quartiles. Length of the box represents the interquartile range, within which 50% of values are located. In pairwise comparisons, the ATT value and CAP M or XL-probe value for each steatosis grade differed significantly from each other (S0 vs. S1, P<0.05; S0 vs. S2, P<0.001).ATT, attenuation coefficient; CAP, controlled attenuation parameter.

The median (95% confidence interval) ATT values (dB/cm/MHz) for each steatosis grade (determined by histological examination of the liver biopsy) was as follows (Fig 3A): S0, 0.57 (0.54–0.60); S1, 0.63 (0.62–0.72); S2, 0.72 (0.56–0.76); and S3, 0.87 (0.74–0.97). ATT values were significantly different between each histological steatosis grade: S0 *versus* S1–3, P<0.001; S0–1 *versus* S2–3, P = 0.0007; and S0–2 *versus* S3, P<0.001. The median liver steatosis values (dB/m) assessed using CAP M-probe for each steatosis grade, were as follows (Fig 3B): S0, 195 (187–210); S1, 253 (226–268); S2, 270 (220–301); and S3, 337 (273–401). Again, there was a significant difference in the CAP values between each histological steatosis grade: S0 *versus* S1–3, P<0.001; S0–1 *versus* S2–3, P = 0.0004; and S0–2 *versus* S3, P = 0.0009). Fig 3C shows the median (95% confidence interval) liver steatosis values assessed using the CAP XL-probe for each steatosis grade: S0, 207 (197–224); S1, 283 (252–302); S2, 271 (233–329); and S3, 323 (272–378). A significant association was observed between the median CAP XL-probe (dB/m) values and histological steatosis grade (S0 and S1–3, P<0.001; S0–1 and S2–3, P = 0.0006; and S0–2 and S3, P = 0.0026). The κ value of S0 or S ≥1 was excellent for both ATT and CAP, with a mean κ-value of 0.91±0.06 for ATT and 0.86±0.08 for CAP.

### Measurement difference between ATT and CAP

ATT was measurable in all cases. In contrast, CAP with the M-probe was unmeasurable in five cases. In these five cases, the subcutaneous fat thickness was >30 mm. Of these five cases, measurements could be recorded using the XL-probe in four cases. However, in the remaining one case, the measurement success rate was <60% even with the XL-probe, and this was not considered a reliable measurement.

### AUC-ROC for the diagnosis of steatosis by ATT and CAP

**Fig 4:**
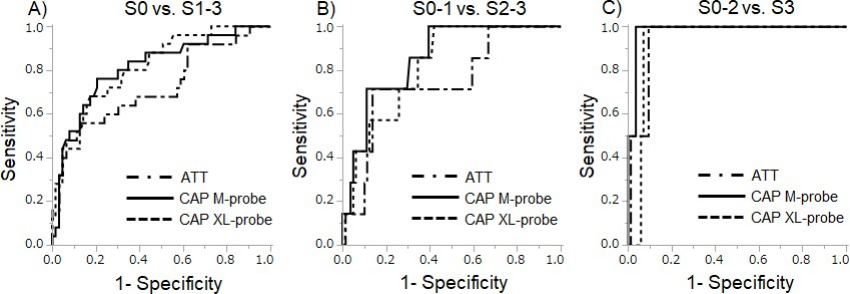
Receiver operating characteristic (ROC) curves for predicting steatosis. ROC curves for diagnosis of mild steatosis (A; S ≥ 1), significant steatosis (B; S ≥ 2), and severe steatosis (C; S = 3). No significant differences in AUC-ROC were found between ATT and CAP examined using the DeLong test. ATT, attenuation coefficient; CAP, controlled attenuation parameter.

The AUC-ROCs for the diagnosis of steatosis using ATT and CAP are shown in Fig 4. AUC-ROCs were 0.74, 0.81 and 0.83 for diagnosing S ≥1 using ATT, CAP with the M-probe and CAP with the XL-probe, respectively, and 0.96, 0.98 and 0.91, respectively, for diagnosing S ≥2, and 0.96, 0.98 and 0.91, respectively, for diagnosing S = 3 (Table 2). ATT cutoffs for S ≥ 1, S ≥ 2, and S = 3, calculated from the AUC-ROC, were 0.68, 0.72 and 0.78 dB/cm/MHz, respectively. For the CAP M-probe, the respective cut-offs were 230, 270 and 324 dB/m, and 267, 230 and 290 dB/m for CAP XL-probe. There was no significant difference between the AUC-ROCs for ATT and CAP in the diagnosis of a steatosis grade ≥ 1 (ATT *versus* CAP M-probe, P = 0.125; and ATT *versus* CAP XL-probe, P = 0.126). Similarly, no significant difference was evident between AUC-ROCs of ATT and CAP in the diagnosis of a steatosis grade ≥ 2 (ATT *versus* CAP M-probe, P = 0.286; and ATT *versus* CAP XL-probe, P = 0.287) or a steatosis grade = 3 (ATT *versus* CAP M-probe, P = 0.570; and ATT *versus* CAP XL-probe, P = 0.831).

**Table 2:** Results of comparisons between ATT and CAP.

Method	S ≥ 1			S ≥ 2			S = 3		
	Sensitivity (%)	Specificity (%)	AUC-ROC	Sensitivity (%)	Specificity (%)	AUC-ROC	Sensitivity (%)	Specificity (%)	AUC-ROC
ATT	55.1	87.7	0.74	77.8	87.1	0.80	100	91.1	0.96
CAP M-probe	76.9	79.4	0.81	71.4	89.0	0.85	100	96.6	0.98
CAP XL-probe	71.4	82.8	0.83	88.9	59.0	0.83	100	80.7	0.91

AUC-ROC, area under the receiver operating characteristic curve; ATT, attenuation coefficient; CAP, controlled attenuation parameter.

The sensitivity for diagnosis of S ≥1 by ATT, CAP M-probe and CAP XL-probe, respectively, were 55.1%, 76.9% and 71.4%, with a specificity of 87.7%, 79.4% and 82.8%, respectively. The sensitivity for S ≥ 2 was 77.8%, 71.4%, and 86.9%, respectively, with a specificity of 87.1%, 89.0%, and 59.0%, respectively. The sensitivity for S = 3 was 100% for ATT, CAP M-probe and CAP XL-probe, with a specificity of 91.1%, 96.6%, and 80.7%, respectively.

### Correlation between ATT and CAP

**Fig 5:**
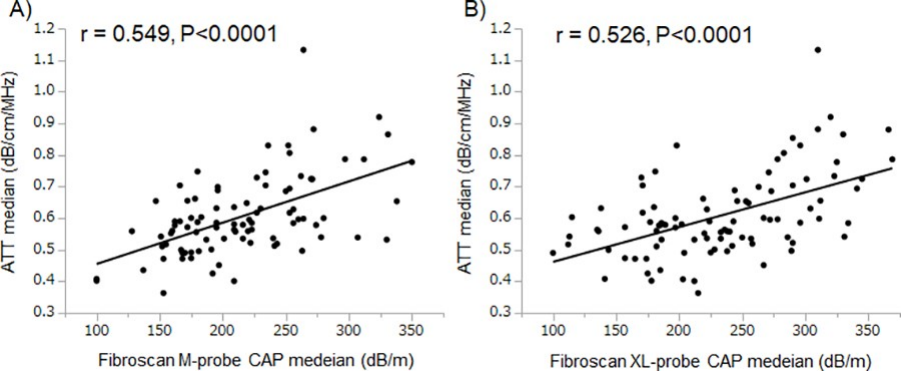
Correlation between ATT and CAP. ATT measurements were significantly correlated with CAP M-probe measurements (r = 0.549, P<0.0001) and CAP XL-probe measurements (r = 0.526, P<0.0001). ATT, attenuation coefficient; CAP, controlled attenuation parameter.

The correlation between the ATT and CAP M-probe value was significant (r = 0.549, P<0.0001, Fig 5A), as was the correlation between the ATT and CAP XL-probe value (r = 0.526, P<0.0001, Fig 5B).

### Measurement time and success rate in each method

**Fig 6:**
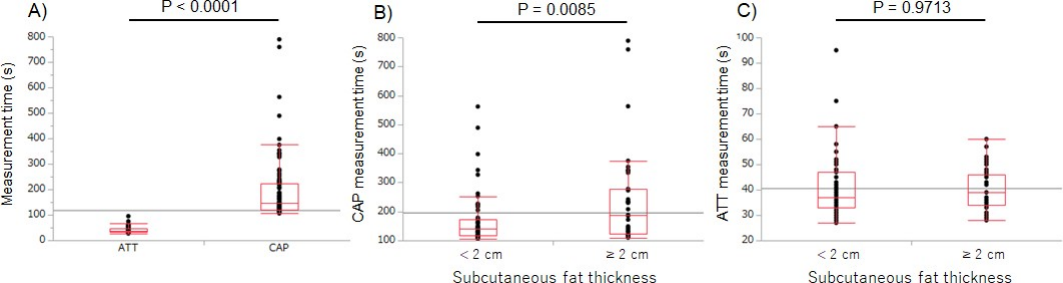
Measurement times for CAP and ATT. A) Measurement time is significantly shorter for ATT than for CAP M-probe (P<0.0001). Measurement times for CAP M-probe (B) and ATT (C), grouped by subcutaneous fat thickness, showing a significantly increased CAP M-probe measurement time when the subcutaneous fat thickness is >2 cm (P = 0.0085), with no effect of subcutaneous fat thickness on ATT measurements (P = 0.9713).

ATT was successfully measured in all cases (success rate, 100%). The mean measurement time for ATT was 40.6±10.9 s. By contrast, CAP measurement was successfully performed in 89 of the 94 cases (success rate, 94.7%), with a mean measurement time of 196.4±128.1 s (which includes the time for cases in which CAP was not successfully measured). Although there was a wide variability in measurement time for both ATT and CAP, overall, the measurement time was significantly shorter for ATT than CAP (P<0.0001, Fig 6A). CAP measurement time was significantly extended in cases where the subcutaneous fat thickness was >2 cm (P = 0.0085, Fig 6B). In contrast, the measurement time for ATT was unaffected by subcutaneous fat thickness (P = 0.9713, Fig 6C).

### Factors affecting ATT measurement

**Fig 7:**
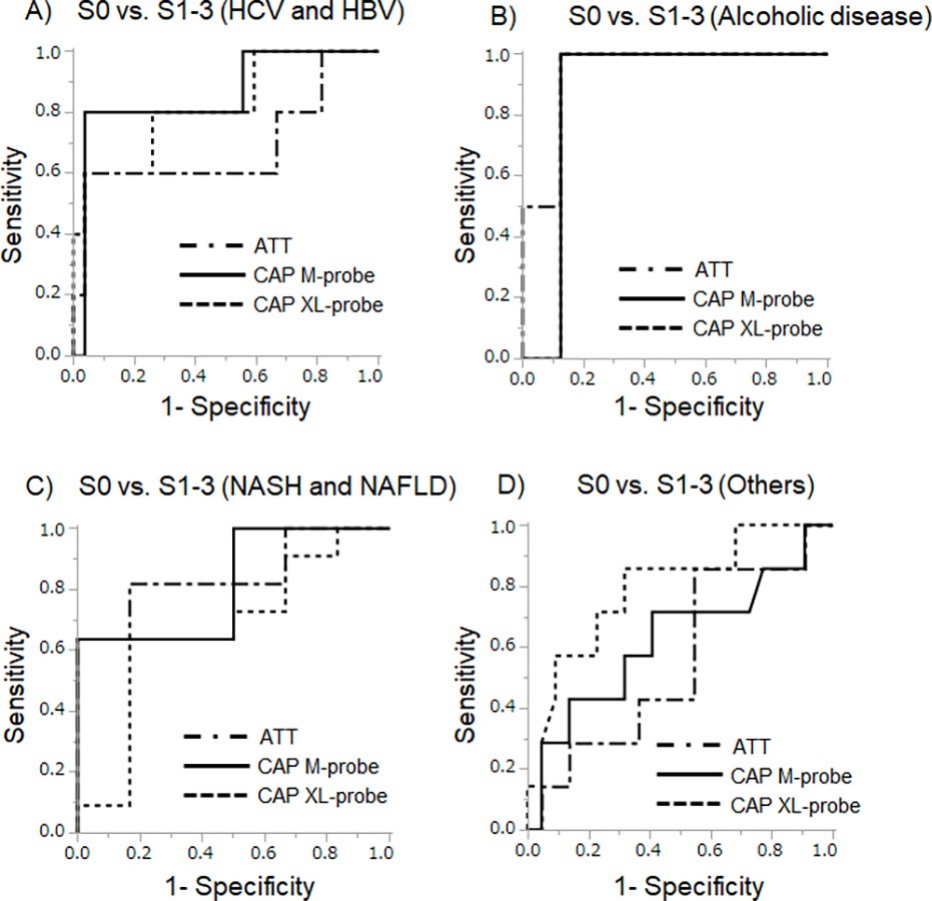
Receiver operating characteristic (ROC) curves by liver etiology. A) ROC curves for hepatitis C virus (HCV)-positive patients and hepatitis B virus (HBV)-positive patients. B) ROC curves for alcoholic liver disease patients. C) ROC curves for nonalcoholic fatty liver disease (NAFLD) patients and nonalcoholic steatohepatitis (NASH) patients. D) ROC curves for other liver disease patients. The AUC-ROC results for each background liver disease varied.

The median ATT value (dB/cm/MHz) for each fibrosis stage was as follows: F0, 0.51; F1, 0.59; F2, 0.66; F3, 0.63; and F4, 0.63. The median ATT value (dB/cm/MHz) for each activity grade was as follows: A0, 0.50; A1, 0.60; A2, 0.64; and A3, 0.74. There was no correlation between the ATT value and either the fibrosis stage and activity grade. However, the ATT value did increase as a function of increasing BMI (Spearman's ρ = 0.31, p = 0.0032). Furthermore, the AUC-ROCs for the diagnosis of steatosis using ATT and CAP by liver etiology are shown in Fig 7. The AUC-ROCs for diagnosing S ≥1 using ATT, CAP with the M-probe, and CAP with the XL-probe were 0.69, 0.86, and 0.82, respectively, in patients with HBV and HCV; 0.94, 0.88, and 0.88, respectively, in those with alcoholic liver disease; 0.84, 0.81, and 0.66, respectively, in those with NASH and NAFLD; and 0.56, 0.63, and 0.80, respectively, in those with other liver diseases.

## Discussion

Ultrasound B mode is a simple and useful method for diagnosing fatty liver, but is limited by the inability to determine, in real time, if only liver parenchyma is included in the measurement [22]. As a solution to this problem, the usefulness of a scoring system adopting the findings of ultrasound B mode images has been reported [7]. The use of CAP as a non-invasive assessment of hepatic steatosis has been proposed [13–17]. However, CAP measurements are influenced by subcutaneous fat thickness, with an XL-probe required in obese patients [23]. Since the transmission frequencies for these two probes differ, numerical values also differ and, thus, values obtained by the M-probe and XL-probe cannot be directly compared. As such, a distinct advantage of ATT is that measurements of hepatic steatosis can be obtained with the same probe, regardless of subcutaneous tissue thickness. Moreover, ATT allows for real time visualization of the target region of measurement, with no additional equipment required. Of note, Jung et al. [24] reported that CAP was unaffected by the state of liver inflammation or liver fibrosis, confirming the utility of CAP for diagnosing steatosis independently of the disease stage or inflammatory activity. Furthermore, in the present study, ATT was measurable in a case (not included in the analysis target) wherein reliable results were not obtained by CAP measurement using the XL-probe. In our own work, we reported similar results for ATT, noting a correlation between ATT values and the grade of fibrosis or inflammatory activity determined by histology [18]. Moreover, ATT values also correlated with the hepatic fat content in the target area of measurement, further underlining the usefulness of ATT for the diagnosis of liver steatosis [18].

In the present study, ATT performed well as a noninvasive method for quantifying hepatic steatosis, providing a good diagnostic accuracy for hepatic steatosis, with AUC-ROCs of 0.80 and 0.96 for steatosis S ≥ 2 and S = 3, respectively. Furthermore, ATT was successfully performed in all cases, and required a significantly shorter time than CAP. Of clinical importance, both ATT and CAP measurements showed high inter-observer agreement.

ATT performance was relatively lower for the diagnosis of S ≥ 1, with an AUC-ROC of 0.74. This finding is consistent with a previous report for CAP, in which the AUC-ROCs ranged from 0.79 for the diagnosis of S1, and increasing to 0.84 for the diagnosis of S2 and S3 steatosis [14]. Similarly, other studies have also reported on the better diagnostic performance of CAP for more severe steatosis grades [25–29]. We do note that two studies reported the diagnostic performance of CAP as being suboptimal for severe steatosis, and further considered the differentiation of steatosis grades 0 and 1 by CAP as being unsatisfactory [30,31]. Diagnosis of mild liver steatosis is important for delineating individuals at risk for NASH and subsequent hepatocellular carcinoma. Further studies are needed to establish optimal diagnostic methods for mild liver steatosis (S<1).

There are several important limitations of the study. First, the number of cases was limited. In particular, few cases of significant or severe liver steatosis (S2 or S3) were included, such that the sample size for S2 and S3 diagnosis may have been insufficient. As a result, the S3 ATT and CAP AUROC (0.96 and 0.98, respectively) and the S1 and S2 AUROC are not comparable (0.74 *versus* 0. 81 and 0.80 *versus* 0. 85, respectively). Third, the target cases in this study had mixed etiologies of liver steatosis. In addition, the AUC-ROC results for each background liver disease varied. Therefore, ATT measurements might be affected by liver etiology. Investigation in a greater number of cases is thus needed to verify the diagnostic accuracy of ATT. We also note that, although we determined inter-observer agreement in measurement, we did not evaluate the variability in inter- and intra-observer measurement for either ATT or CAP.

In conclusion, measurements obtained with the newly developed ATT correlated with CAP values. ATT offers distinct advantages over CAP; hence, it is potentially more clinically useful than CAP. Analysis using ATT allows real time visualization without the requirement for special equipment; ATT measurement is simple and quick to perform and is are not affected by subcutaneous tissue thickness. Therefore, ATT should be considered for the assessment of liver steatosis, in particular, when CAP measurements cannot be obtained.

## Supporting information

S1 Checklist TREND checklist. (PDF)

S1 Dataset Sets of data, median value of ATT and CAP. (XLSX)

S2 Dataset Sets of data, measurement time of ATT and CAP. (XLSX)

S1 Protocol Trial study protocol in original language (Japanese). (DOC)

S2 Protocol Translated trial study protocol. (DOCX)
