# Supplementary material for: New diagnostic technique to evaluate hepatic steatosis using the attenuation coefficient on ultrasound B mode
Source: PLoS One. 2019 Aug 27;14(8):e0221548. doi: 10.1371/journal.pone.0221548 (PMC6711590; doi:10.1371/journal.pone.0221548)
Supplement: S1 Protocol — (DOC) [file pone.0221548.s004.doc]

**超音波による非侵襲的肝機能総合評価技術の有用性に**

**ついての研究**

**―　研究計画書　―**

## **機密保持に関するお願い**

## 本計画書は、本研究運営委員会の知的所有物です。従って、本研究会からの文書による事前の許可なく、第三者に本研究計画書に関する情報を開示または漏洩しないようお願いいたします。

**2015年　06月　２9日　初版作成**

**目　次**

　ページ

1. 試験の概要　・・・・・・・・・・・・・・・・・・・・・・・・・・・・・　3
2. 目的　・・・・・・・・・・・・・・・・・・・・・・・・・・・・・・・・　5
3. 背景・・・・・・・・・・・・・・・・・・・・・・・・・・・・・・・・・　5
4. 対象・・・・・・・・・・・・・・・・・・・・・・・・・・・・・・・・・　7
5. 試験計画・・・・・・・・・・・・・・・・・・・・・・・・・・・・・・・　8
6. 調査および検査　・・・・・・・・・・・・・・・・・・・・・・・・・・・　11
7. 有害事象の評価と報告　・・・・・・・・・・・・・・・・・・・・・・・・　15
8. データ収集・・・・・・・・・・・・・・・・・・・・・・・・・・・・・・　17
9. エンドポイント・・・・・・・・・・・・・・・・・・・・・・・・・・・・　18
10. 統計学的事項　・・・・・・・・・・・・・・・・・・・・・・・・・・・・　18
11. 倫理・・・・・・・・・・・・・・・・・・・・・・・・・・・・・・・・・　20
12. 品質管理・品質保証・・・・・・・・・・・・・・・・・・・・・・・・・・　22
13. 記録の保存　・・・・・・・・・・・・・・・・・・・・・・・・・・・・・　22
14. 個人個人情報の管理と匿名化の方法・・・・・・・・・・・・・・・・・・・　22
15. 研究成果の発表　・・・・・・・・・・・・・・・・・・・・・・・・・・・　23
16. 臨床試験登録　・・・・・・・・・・・・・・・・・・・・・・・・・・・・　23
17. 利益相反と研究資金源・・・・・・・・・・・・・・・・・・・・・・・・・　24
18. 試験参加者の費用と健康被害への対応　・・・・・・・・・・・・・・・・・　24
19. 試験実施計画の遵守、変更・・・・・・・・・・・・・・・・・・・・・・・　25
20. 研究組織・・・・・・・・・・・・・・・・・・・・・・・・・・・・・・・　27
21. 試験実施施設　・・・・・・・・・・・・・・・・・・・・・・・・・・・・　28
22. 参考文献　・・・・・・・・・・・・・・・・・・・・・・・・・・・・・・　30

**試験の概要**

- 1. 試験計画

肝生検あるいは肝切除を受ける肝疾患患者を対象としたエラストグラフィ及び減衰計測による肝機能評価に関する研究

肝生検あるいは肝切除前後4ヶ月以内に血液検査、Real-time Tissue Elastography (RTE)、Shear Wave Measurement (SWM)、減衰計測、FibroScan、Virtual Touch Quantification (VTQ)、ShearWave Elastography (SWE)、Shear wave with Smart maps (SwSm)、Shear Wave Elastography (SWE_GE)、MR Touchを施行

肝生検あるいは肝切除を受ける肝疾患患者

- 1. 目的

肝生検あるいは肝切除を受ける肝疾患患者に対し、Real-time Tissue Elastography（以下RTE）、Shear Wave Measurement（以下SWM）、減衰計測を実施し、それらを用いた肝臓の線維化、炎症、脂肪化の指標を推定するためのアルゴリズムを開発する。

同時に、それらの指標と肝組織診断、血液検査、または他のエラストグラフィ（FibroScan、Virtual Touch Quantification (VTQ)、ShearWave Elastography (SWE)、Shear wave with Smart maps (SwSm)、Shear Wave Elastography (SWE_GE)、MR Touch）とを比較し、肝臓の線維化、炎症、脂肪化など肝臓の病態全般を非侵襲的に評価しうるかどうか検討する。

- 1. 評価項目
     1. 主要評価項目Primary Endpoint
- RTE、SWM、減衰計測を用いて肝臓の線維化、炎症、脂肪化の指標を算出するためのアルゴリズムを開発する。
  - 1. 副次的評価項目Secondary Endpoints
- RTE、SWM、減衰計測を用いて算出した指標と血液検査結果との関連性
- RTE、SWM、減衰計測を用いて算出した指標と病理学的診断との相関性
- RTE、SWM、減衰計測を用いて算出した指標と他のエラストグラフィとの相関性
  1. 対象
     1. 対象者

①あるいは②に該当するものを対象とする。

①肝生検あるいは肝切除を受ける肝疾患患者

②健常ボランティア

血清学的あるいは画像診断学的に肝疾患を否定できる症例を健常者とする。

- - 1. 選択基準

指定がない限り、検査および所見は登録前6ヶ月以内の結果を用いる。

1. 年齢・性別：20才以上の男女
2. 本試験への参加にあたり十分な説明を受けた後、十分な理解の上、患者本人の自由意思による文書同意が得られた患者

〔選択基準の設定根拠〕

1. 本人からの同意取得が可能な20歳以上を対象とした。
2. ヘルシンキ宣言に基づく。
   - 1. 除外基準
3. エラストグラフィのROI（関心領域Region of Interest）を腫瘍から外して測定できない患者
4. 妊婦および妊娠している可能性のある患者、または授乳中の患者
5. その他、研究担当医師が不適切と判断した患者

〔除外基準の設定根拠〕

(1) 適切に非腫瘍部のエラストグラフィが測定できない症例を除外した。

(2)～(3)安全性を考慮した。

- 1. 目標被検者数と試験実施予定機関
- 目標被検者数：

450名

- - 1. 400名：生検・手術症例
    2. 50名：健常者ボランティア
- 登録期間：倫理委員会承認後から平成2９年3月31日まで。ただし、必要に応じて期間を短縮または延長する。
- 試験実施期間：倫理委員会承認後から最終の患者登録後3ヶ月後まで。ただし、必要に応じて期間を短縮または延長する。
  1. 問い合わせ先

研究事務局

近畿大学医学部　消化器内科　矢田　典久

〒589-8511　大阪府大阪狭山市大野東377-2

TEL : 072-366-0221（内線 3525）、FAX : 072-368-2880

E-mail : yada@med.kindai.ac.jp

1. **目的**

肝生検あるいは肝切除を受ける肝疾患患者に対し、Real-time Tissue Elastography（以下RTE）、Shear Wave Measurement（以下SWM）、減衰計測を実施し、それらを用いた肝臓の線維化、炎症、脂肪化の指標を推定するためのアルゴリズムを開発する。

同時に、それらの指標と肝組織診断、血液検査、または他のエラストグラフィ（FibroScan、Virtual Touch Quantification (VTQ)、ShearWave Elastography (SWE)、Shear wave with Smart maps (SwSm)、Shear Wave Elastography (SWE_GE)、MR Touch）とを比較し、肝臓の線維化、炎症、脂肪化など肝臓の病態全般を非侵襲的に評価しうるかどうか検討する。

1. **背景**
   1. 背景

　HBVあるいはHCVによる慢性肝疾患は、病態が進行するほど発癌リスクが高くなり、また胃食道静脈瘤などの門脈亢進症状、肝不全のリスクが上昇する。また、肝硬変はインターフェロンの治療抵抗性宿主因子の1つとしても知られている。これらの慢性肝疾患の治療を行うためには、肝臓の線維化、炎症、及び脂肪化を評価し、病態を把握することが重要である。

　肝臓の線維化、炎症、及び脂肪化は、肝生検で調べるのが確定診断としては重要であるが、侵襲的でありサンプリング・エラーが生じる可能性もある。

線維化においては、非侵襲的な指標として、各種血清マーカーが多数報告されている。また、近年、超音波技術を用いて肝硬度を測定する装置が様々開発され、肝硬度と線維化の程度との良好な相関性が得られていることが多数報告されている。特にプローブから発振される振動の伝達速度を検出して肝組織の剪断弾性率を算出するFibroScanに関する報告は数多く認め、良好な結果が得られている。一方、RTEは手圧迫や振動による組織の歪み分布をリアルタイムに映像化でき、乳腺腫瘍や前立腺腫瘍、甲状腺腫瘍での有効性が報告されている[14-16]。同様に肝臓においても心拍による歪み画像から線維化度の測定に有用である可能性が少数ながら報告されている。FibroScanや血清マーカーよりも有用であるとの報告もあるが、まだ適切な評価方法は定まっていない。また、音響放射圧を利用して軟部組織を微小変位させるというAcoustic Radiation Force Impulse (ARFI)という技術により発生させて生じた組織の歪み（剪断）が元に戻る際に発生する剪断波の伝播速度を測定するVirtural Touch Quantification（以下VTQ）やShearWave Elastography（以下SWE）、Shear wave with Smart maps（以下SwSm）、Shear Wave Elastography（以下SWE_GE）は、肝硬度測定ツールとして有用であるとの多数の報告が行われている。さらに近年、MRIを用いて肝剛性を測定するMR Elastographyも開発され、MR Touchが臨床応用されはじめた。

FibroScan、VTQ、SWE、SwSm、SWE_GEといったShear wave imagingによって測定される肝硬度は、線維化以外に炎症・黄疸・鬱血などの影響を受け[10,20-22]、一方RTEは炎症の影響を受けずに線維化の評価ができるとの報告もある。したがって、両者を同時に測定することで、肝臓の炎症と線維化の程度を分けて評価することができる可能性がある。

一方、肝臓の脂肪化については、超音波の減衰係数から求める方法が1980年代より報告されており[24-27]、FibroScanで肝硬度を測定するのと同時に超音波の減衰係数を求めるControlled Attenuation Parameter(CAP)が搭載され、その有用性が報告されている[28]。

今回、日立アロカメディカル株式会社からShear wave elastography であるSWMと減衰計測機能が開発され、従来のStrain elastographyであるRTEと同一の装置上で測定できるようになった。

そこで、肝生検あるいは肝切除を受ける肝疾患症例を対象とし、病理学的診断結果を教師データとしてRTE、SWM及び減衰計測の結果を用いて肝臓の線維化、炎症、脂肪化の指標を算出するアルゴリズムを検討する。また、採血検査、病理学的診断、FibroScan、VTQ、SWE、SwSm、SWE_GE及びMR Touchを行い、これらの相関性を評価するとともに、その有用性について検討する。

1. **対象**
   1. 対象者

①あるいは②に該当するものを対象とする。

①肝生検あるいは肝切除を受ける肝疾患患者

②健常ボランティア

血清学的あるいは画像診断学的に肝疾患を否定できる症例を健常者とする。

- 1. 選択基準

指定がない限り、検査および所見は登録前6ヶ月以内の結果を用いる。

1. 年齢・性別：20才以上の男女
2. 本試験への参加にあたり十分な説明を受けた後、十分な理解の上、患者本人の自由意思による文書同意が得られた患者

〔選択基準の設定根拠〕

1. 本人からの同意取得が可能な20歳以上を対象とした。
2. ヘルシンキ宣言に基づく。

- 1. 除外基準

1. エラストグラフィのROI（関心領域Region of Interest）を腫瘍から外して測定できない患者
2. 妊婦および妊娠している可能性のある患者、または授乳中の患者
3. その他、研究担当医師が不適切と判断した患者

〔除外基準の設定根拠〕

(1) 適切に非腫瘍部のエラストグラフィが測定できない症例を除外した。

(2)～(3)安全性を考慮した。

**試験計画**

- 1. 試験デザイン

多施設共同横断試験

- - 1. 試験全体のアウトライン
- 肝生検あるいは肝切除前後4ヶ月以内に、採血、超音波エラストグラフィ（RTE、SWM、FibroScan、VTQ、SWE、SｗSm、SWE_GE）、減衰計測、MR Touchを施行する。
- 超音波エラストグラフィ及び減衰計測施行と同日に採血を行う。

医師の診断

適格基準の確認

同意取得

登録日

採血

エラストグラフィ、減衰計測

肝生検

肝切除術

肝生検あるいは肝切除

前後4ヶ月以内

エラストグラフィ : SWM、RTE、FibroScan、VTQ、SWE、SｗSm、SWE_GE、MR Touch

- - 1. 目標被検者数と試験実施予定期間
- 目標被検者数：

450名

- - 1. 400名：生検・手術症例
    2. 50名：健常者ボランティア
- 登録期間：倫理委員会承認後から平成2９年3月31日まで。ただし、必要に応じて期間を短縮または延長する。
- 試験実施期間：倫理委員会承認後から最終の患者登録後3ヶ月後まで。ただし、必要に応じて期間を短縮または延長する。
  1. 登録
  2. 担当医師は、適格条件を満たす患者に対し本研究について説明し、同意を取得する（10.1）。
  3. 担当医師は、登録に同意した患者について、選択基準をすべて満たし、除外基準のいずれにも該当しないことを確認し、「症例登録票」に必要事項をすべて記入の上、データセンターに「症例登録票」をFAX送信する。
  4. データセンターは、症例登録票の内容および適格性を確認し、不備があればその内容を担当医師に確認の上、すべての基準が満たされていることを確認した後、登録を受け付ける。
  5. データセンターは、適格・不適格の結果を「症例登録票」に記載された担当医師のFAX番号宛にFAX送信する。適格であれば、症例登録番号が記載された「登録確認書」を発行する。登録確認書のFAX送信をもって「登録完了」とする。不適格であれば、「不適格通知書」を担当医師宛にFAX送信する。

##### 担当医師は、症例登録票の原本および登録確認書を保管する。データセンターはFAXで送付された症例登録票を写しとして保管する。

＜登録先＞

データセンター

担当：弓削 公子

TEL : 072-366-0221（内線 6458）、FAX : 072-368-1903

E-mail : kin-live@med.kindai.ac.jp

＜適格基準に関する問い合わせ先＞

研究事務局

近畿大学医学部　消化器内科　矢田　典久

〒589-8511　大阪府大阪狭山市大野東377-2

TEL : 072-366-0221（内線 3525）、FAX : 072-367-2880

E-mail : yada@med.kindai.ac.jp

- - 1. 登録に際しての注意事項

1. 登録日は、一連の登録手続きが完了した日とし、登録確認書に記載される。データセンターに症例登録票をFAXした時点では「登録」とはならない。
2. データの研究利用の拒否を含む同意撤回があった場合を除き、一度登録された患者は、登録取り消し（データベースから抹消）されない。重複登録の場合は、いかなる場合も初回の登録情報（症例登録番号）を採用する。
3. 誤登録・重複登録が判明した場合は、速やかにデータセンターに連絡する。
   1. 試験完了の定義

試験治療後、死亡または観察期間終了まで経過観察を完遂した場合を「試験完了」と定義する。死亡または観察期間終了の前に経過観察を中止した場合を「試験中止」とする。

- 1. 試験検査前の中止

担当医師は患者の登録後、登録時に不適格であったことが判明した場合、患者から試験検査中止の申し出があった場合、担当医師が試験治療の実施を不適当と判断した場合などは、試験検査を中止する。試験検査の開始前に試験検査を中止した場合は、中止の理由および詳細についてカルテおよび症例報告書に記録する。

登録後の同意の撤回があった場合は、担当医師はその理由をできるだけ詳細に明らかにし、有効性・安全性の評価対象症例の採否の参考となるように症例報告書に記録すること。

- 1. 併用療法
     1. 併用禁止療法

併用禁止薬を設けない。

- 1. 検査後の経過観察

検査後、30日間は試験に伴う副作用がなかったかを経過観察する。

1. **調査および検査**
   1. 登録時の調査
      1. 患者背景

生年月日、年齢、性別、身長、体重、腹囲、体表から肝実質までの厚さ

- 腹囲の測定方法は、メタボリック・シンドローム診断と同様に立位、軽呼気時、臍周囲で測定するが、脂肪蓄積が著明で臍が下方に偏位している場合は肋骨下縁と前上腸骨棘の中点の高さで測定する。

PS (performance status)

肝切除目的（原発性肝癌、転移性肝癌、その他）

背景肝（C型肝炎、B型肝炎、アルコール性肝障害、非アルコール性脂肪性肝疾患など）

アルコール摂取量・期間

抗ウイルス治療の有無・内容

胸水・腹水の有無・程度

不整脈の有無・内容

心疾患の有無・内容

その他の既往歴・合併症

- 1. 登録後調査
- 肝生検あるいは肝切除前後4ヶ月以内に、採血、超音波エラストグラフィ（RTE、SWM、FibroScan、VTQ、SWE、SｗSm、SWE_GE）、減衰計測、MR Touchを施行する。
- 超音波エラストグラフィ及び減衰計測施行と同日に採血を行う。
  - 1. 血液検査

下記の検査の内、下線（　　）のものは、空腹時に採血を行う。

生化学：AST、ALT、γGTP、ALP、総ビリルビン、直接ビリルビン、総蛋白、アルブミン、タンパク分画、コリンエステラーゼ、総コレステロール、LDLコレステロール、HDLコレステロール、中性脂肪、BUN、Cr、血糖、HbA1C（NGSP）、インスリン、フェリチン、血清Fe、TIBC、UIBC

血球　：白血球数、赤血球数、ヘモグロビン、ヘマトクリット、血小板数

凝固　：プロトロンビン時間（%、INR）

線維化マーカー：ヒアルロン酸、Ⅳ型コラーゲン7S、プロコラーゲンⅢペプチド（P-Ⅲ-P）、M2BPGi

また、基礎疾患の鑑別用の検査値として下記の検査を行う。

（6ヶ月以内に行ったものがあれば、新たに検査せずに代用できるものとする。）

　HCV：HCV抗体、HCV-RNA量（HCV抗体陽性の場合）

　HBV：HBs抗原、HBs抗体、HBc抗体、HBV-DNA量（HBs抗原陽性の場合）

　原発性胆汁性肝硬変：抗ミトコンドリア抗体（あるいは抗ミトコンドリアM2抗体）、IgM

　自己免疫性肝炎：抗核抗体、IgG

- - 1. 肝病理組織診断
- 肝生検および肝切除病理標本は、①H.E.染色および②Masson trichrome染色する。
- プレパラートをバーチャルスライド化し、独立病理読影委員に送付する。
- 独立病理読影委員は、新犬山分類に従い線維化を評価する。
  - 1. Real-time Tissue Elastography® （RTE）
- 使用装置：日立アロカメディカル（株）Ascendus
- 探触子：コンベックスプローブEUP-C715
- 測定方法の詳細については、別紙（RTE撮像方法）に示す。
- データは、匿名化したうえで日立アロカメディカル㈱に送付し、日立アロカメディカル㈱にて患者背景をブラインドにした状態で解析する。
  - 1. Shear Wave Measurement（SWM）
- 使用装置：日立アロカメディカル（株）Ascendus
- 探触子：コンベックスプローブEUP-C715
- 測定方法の詳細については、別紙（SWM撮像方法）に示す。
- データは、匿名化したうえで日立アロカメディカル㈱に送付し、日立アロカメディカル㈱にて患者背景をブラインドにした状態で解析する。
  - 1. 減衰計測機能
- 使用装置：日立アロカメディカル（株）Ascendus
- 探触子：コンベックスプローブEUP-C715
- 測定方法の詳細については、別紙（減衰計測撮像方法）に示す。
- データは、匿名化したうえで日立アロカメディカル㈱に送付し、日立アロカメディカル㈱にて患者背景をブラインドにした状態で解析する。
  - 1. Vibration-Controlled Transient Elastography（VCTE）
- FibroScanを所有している施設に関しては、肝生検、肝切除、RTE、またはSWMによる肝線維化評価の前後4ヶ月以内にVibration-Controlled Transient Elastographyでの評価も行う。
- 探触子：M-probe、XL-probe
- 右肋間にて10回検査し、60%以上の有効性が認められ、IQRが測定値の1/3未満の際にその検査を有効と判断する。
  - 1. Controlled Attenuation Parameter (CAP)
- FibroScanを所有している施設に関しては、肝生検、肝切除、や減衰計測機能による肝脂肪化評価の前後4ヶ月以内にCAPでの評価も行う。
- 探触子：M-probe、XL-probe
- 右肋間にて10回検査し、60%以上の有効性が認められ、IQRが測定値の1/3未満の際にその検査を有効と判断する。
  - 1. Virtual touch quantification (VTQ)
- ACUSON S2000を所有している施設に関しては、肝生検、肝切除、RTE、またはSWMによる肝線維化評価の前後4ヶ月以内にVTQでの評価も行う。
- 使用装置：持田SIEMENS社 ACUSON S2000
- 探触子：4C1
- 右肋間操作にて肝右葉を描出し、ROIを肝表面から1-4cm下に置き、5回検査し中央値を算出する。
- 測定の際、肝表面はエコー画面に対して出来るだけ水平（プローブと平行）にする。
  - 1. ShearWave Elastography (SWE)
- Aixplorerを所有している施設に関しては、肝生検、肝切除、RTE、またはSWMによる肝線維化評価の前後4ヶ月以内にSWEでの評価も行う。
- 使用装置：コニカミノルタ Aixplorer
- 探触子：SC6-1
- 右肋間操作にて肝右葉を描出し、多重反射の影響を受けないようにROIを肝表面から数cm下に置き、5回検査し中央値を算出する。
- 測定の際、肝表面はエコー画面に対して出来るだけ水平（プローブと平行）にする。
  - 1. Shear wave with Smart maps (SwSm)
- Aplio500 Platinumを所有している施設に関しては、肝生検、肝切除、RTEまたはSWMによる肝線維化評価の前後4ヶ月以内にSwSmでの評価も行う。
- 使用装置：東芝 Aplio500 Platinum
- 探触子：375BT
- 右肋間操作にて肝右葉を描出し、多重反射の影響を受けないようにROIを肝表面から数cm下に置き、5回検査し中央値を算出する。
- 測定の際、肝表面はエコー画面に対して出来るだけ水平（プローブと平行）にする。
  - 1. MR Touch
- MR Touchを所有している施設に関しては、肝生検、肝切除、RTE、またはSWMによる肝線維化評価の前後4ヶ月以内にMR Touchでの評価も行う。
- 使用装置：GEヘルスケアジャパン(株) MR Touch対応機種
  - 1. Shear Wave Elastography (SWE_GE)
- LOGIQ E9を所有している施設に関しては、肝生検、肝切除、RTE、またはSWMによる肝線維化評価の前後4ヶ月以内にSWE_GEでの評価も行う。
- 使用装置：GE LOGIQ E9
- 探触子：C1-6-DもしくはC1-6VN-Dプローブ
- 右肋間操作にて肝右葉を描出し、多重反射の影響を受けないようにROIを肝表面から数cm下に置き、5回検査し中央値を算出する。
- 測定の際、肝表面はエコー画面に対して出来るだけ水平（プローブと平行）にする。

1. **有害事象の評価と報告**

有害事象とは、被験者に発現したあらゆる好ましくない症状および徴候（臨床検査値の異常を含む）と定義し、試験治療との因果関係の有無を問わない。有害事象が発現した場合、担当医師は速やかに必要な対応（検査、治療、試験中止など）を行い、被験者の安全の確保に努める。また、以下に定める手順に従い有害事象の評価および報告を行う。

- 1. 有害事象の評価
     1. 評価対象有害事象

本試験では、試験治療開始以降～試験検査後30日以内に発現した重篤な有害事象のうち、試験検査との関連が否定されないものを評価対象とする。ただし、原疾患の悪化は除く。

- - 1. 調査項目

担当医師は評価対象有害事象の発現の有無を調査する。以下の項目を調査し、カルテおよび症例報告書に記入する。

事象名（原則として診断名）、発現日、重篤度（重篤、非重篤）、処置の有無、程度（軽度、中等度、重度）、経過（消失、軽減、不変、悪化を観察した日）、試験検査との因果関係（関連あり、たぶん関連あり、おそらく関連なし、関連なし）およびその判定理由

- - 1. 重篤な有害事象

以下のいずれかに該当する場合は重篤な有害事象とする。

#### 死に至るもの

#### 生命を脅かすもの（その事象が起こった際に患者が死の危険にさらされていた場合）

#### 治療のための入院または入院期間の延長が必要となるもの

#### 永続的または顕著な障害・機能不全に陥るもの

#### 先天異常を来すもの

#### その他の医学的に重要な状態（即座に生命を脅かしたり死や入院には至らなくとも、患者を危機にさらしたり、上記のような結果に至らぬように処置を必要とするような重大な事象）

- - 1. 程度

#### 軽度：無処置で試験継続可能な程度あるいは日常生活に支障とならないもの

#### 中等度：処置は必要であるが試験継続可能な程度あるいは日常生活に支障を与えるもの

#### 重度：試験継続不可能な程度（被験者の申し出による中止は除く）あるいは日常生活を不可能にするもの

- - 1. 経過

症例報告書には判定日を記入し、可能な限り「消失」まで経過を追跡する。

#### 消失：症状が消失した場合

#### 軽減：症状が軽減した場合

#### 不変：症状が不変の場合

#### 悪化：症状が悪化した場合

#### 不明

- - 1. 試験検査との因果関係の基準

以下の要因をもとに、試験検査により有害事象が引き起こされたと考えられる合理的可能性の有無について5段階（関連あり、たぶん関連あり、おそらく関連なし、関連なし）で判定する。

合理的可能性の要因

#### 時間経過：当該試験検査と有害事象の発現に合理的時間関係がある。

#### 既知治療特性：当該試験検査の既知情報と一致、または、当該試験検査の特性から予測し得る。

#### 他の原因の存在：他の理由に加え、他の治療、原疾患、基礎疾患、宿主要因、環境因子などで合理的説明が出来る。

#### 特定の検査：特定の検査により因果関係が証明される。

- 1. 有害事象の報告

各施設の担当医師は、評価対象有害事象（試験治療開始以降～試験治療後30日以内に発現した重篤な有害事象のうち、試験検査との関連が否定されないもの）を認めた場合、速やかに重篤有害事象緊急報告書により速やかにデータセンターへFAX送信する。データセンターは評価対象有害事象の発生について定期的に研究代表者に報告する。研究代表者は評価対象有害事象の発生について研究グループおよび試験実施施設と情報共有する。

- - 1. 独立データモニタリング委員会への報告

#### 研究代表者は、各施設から報告された評価対象有害事象について定期的に独立データモニタリング委員会に文書で報告し、同時に当該有害事象に対する研究代表者の見解と有害事象に対する対応の妥当性についての審査を依頼する。

#### 独立データモニタリング委員会は、報告内容を審査し、症例の取り扱いや登録継続の可否を含む今後の対応について研究代表者に文書で勧告する。

1. **データ収集**
   1. 症例報告書（case report form、CRF）
      1. データの提出

本試験では、症例登録票およびそれ以外の症例報告書データはFAXにより提出する。担当医師または臨床試験コーディネーター（clinical research coordinator、CRC）等は、本試験に登録されたすべての症例を対象として試験完了まで、試験の進捗にあわせてデータセンターにFAX用いてデータの提出を行う。CRC等が記入あるいは入力を行う場合には担当医師の確認を得る。提出するデータの内容、提出の手段および提出時期を以下に示す。

- - 1. 症例報告書の種類と提出期限

本試験で用いるCRFの種類、送付・提出の手段および時期を以下に示す。

| No. | 種類 | 施設への送付手段、時期 | 提出手段、時期 |
| --- | --- | --- | --- |
| 1 | 症例登録票 | 郵送  施設登録後ただちに | FAX送信  登録時 |
| 2 | 症例報告書 | 郵送  施設登録後ただちに | FAX送信 各調査時点より1ヶ月以内 |
| 3 | 重篤有害事象緊急報告書 | 院内書式の使用も可  参加施設へ予め郵送 | FAX送信 緊急報告対象有害事象の発現を知ってから速やかに |

- - 1. データマネジメント

データセンターは、別に定めるデータマネジメント計画（standard operating procedureとマニュアル）に従って、提出されないデータの督促、提出されたデータの精査と問い合わせ、問い合わせ結果に基づくデータ修正、データベース管理を行う。また、データセンターは、モニタリング用の資料および統計解析用データセットを作成する。

# **エンドポイント**

- 1. エンドポイントの定義
     1. Primary endpoint（主要評価項目）
- RTE、SWM、減衰計測を用いて肝臓の線維化、炎症、脂肪化の指標を算出するためのアルゴリズムを開発する
  - 1. Secondary points（副次評価項目）
- RTE、SWM、減衰計測を用いて算出した指標と血液検査結果との関連性
- RTE、SWM、減衰計測を用いて算出した指標と病理学的診断との関連性
- RTE、SWM、減衰計測を用いて算出した指標と他のエラストグラフィ及び減衰計測との相関性

1. **統計学的事項**
   1. 解析対象集団の定義

定期モニタリング、中間解析、最終解析で用いる解析対象集団について以下のように定義する。

#### 全登録例：登録手順に沿って登録されたすべての患者を「全登録例」とする。

#### 不適格例：登録された患者のうち、登録の基準を満たしていない（合致していないことが後から判明した）患者を「不適格例」とする。

#### 全適格例：全登録例から「研究グループで適切に決定した不適格例」を除く集団を「全適格例」とする。

- 1. Primary endpointの解析

病理学的診断とRTE・SWM・減衰計測との関連性について、重回帰分析やデータマイニングなども用いて肝臓の線維化、炎症、脂肪化の指標を算出する。また、疾患ごとにサブ解析も行い、検討する。

- 1. Secondary endpointsの解析

各エラストグラフィ及び減衰計測による肝臓の線維化、炎症、脂肪化の指標と血清マーカーとの関連性を評価するためにPearson相関係数などをそれぞれ求める。

各エラストグラフィ及び減衰計測による肝臓の線維化、炎症、脂肪化の指標と病理学的診断との関連性を評価するためにPearson相関係数などをそれぞれ求める。

各エラストグラフィ及び減衰計測による肝臓の線維化、炎症、脂肪化の指標と他のエラストグラフィ及び減衰計測との相関性を評価するためにPearson相関係数などをそれぞれ求める。

また、疾患ごとにサブ解析も行い、検討する。

- 1. 目標被験者数の設定根拠

肝臓の線維化、炎症、脂肪化の指標を算出するために、教師データとして肝生検あるいは肝切除症例が300例と、健常例が30例必要である。また、算出した肝臓の線維化、炎症、脂肪化の指標の有用性を検証するために、肝生検あるいは肝切除症例が１００例と、健常例が20例必要である。本試験実施施設ではそれぞれ肝生検症例は年間250件、肝切除症例は年間約50件あるため、3施設の合計で400症例の病理学的診断との比較も容易に担保できる予定である。また、健常者ボランティアは各施設で20例撮像できる予定である。

- 1. 付随研究による探索的な解析

本試験のデータを用いた付随研究を新たに提案する場合は、検討したい内容を文書にまとめ、本研究代表者に提出する。研究代表者は、提案のあった付随研究の内容と実施方法について提案者とともに検討する。

1. **倫理**

本試験に関係するすべての研究者はヘルシンキ宣言および厚生労働省「臨床研究に関する倫理指針」（http://www.mhlw.go.jp/stf/seisakunitsuite/bunya/hokabunya/kenkyujigyou

/i-kenkyu/index.html）を遵守して本試験を実施する。

- 1. インフォームド・コンセント
     1. 患者への説明および同意の取得

担当医師は、患者が試験に参加する前に、患者に対し施設の倫理審査委員会（またはInstitutional Review Board、IRB）で承認の得られた説明文書を渡し、口頭で十分に説明する。試験の説明を行った後、質問する機会と判断するのに十分な時間を与え、患者が試験の内容をよく理解したことを確認した上で試験への参加を依頼し、本人の自由意思による同意を同意書で得るものとする。

同意書には、説明を行った担当医師のほか、試験協力者が補足的な説明を行った場合には、当該試験協力者が、各々その日付を記入の上で記名捺印または署名し、被験者は同意日を記入の上で記名捺印または署名する。同意書の写しは被験者に提供し、原本は施設で保存する。

- - 1. 試験への継続参加について被験者の意思に影響を与える可能性のある情報が得られた場合

#### 担当医師は、被験者が試験に参加している間に、本試験への継続参加について被験者の意思に影響を与える可能性のある情報を入手した場合には、直ちに当該情報を記載した説明資料を提供し、これに基づき、以下について被験者に説明する。

###### 当該情報について

###### 本試験への継続参加については自由であること

#### 説明文書・同意書には、説明を行った担当医師が、説明した日付を記入の上で記名捺印または署名し、被験者は情報受領日を記入の上記名捺印または署名する。なお、試験協力者が補足的な説明を行った場合には当該試験協力者もその日付を記入の上で記名捺印または署名する。当該資料の写しは被験者に提供する。担当医師は、本試験に継続して参加するか否かについての意思を被験者に確認し、説明文書・同意書の原本に意思確認日およびその確認結果を記入し、保存する。

#### 担当医師は、説明文書・同意書を改訂し、倫理審査委員会の承認を得た後、被験者に改訂された説明文書・同意書を用いて改めて説明し、本試験への参加の継続について被験者から自由意思による同意を文書により得る。その際、担当医師は、当該同意書に説明日を記入の上で記名捺印または署名し、被験者も同意日を記入の上で記名捺印または署名する。同意書の写しは被験者に提供し、原本を施設で保存する。

- 1. 被験者の人権および安全性・不利益に関する配慮
     1. 人権への配慮（プライバシーの保護）

試験実施に係わる生データ類および同意書等を取扱う際は、被験者の秘密保護に十分配慮する。また、病院外に提出する症例報告書の作成、取扱い等においても、登録割付時に発行される「症例登録番号」と施設内で設定する「被験者識別コード」により被験者を特定し、その秘密保護について配慮する。本試験で得られた被験者のデータは本試験の目的以外には使用しない（本研究以外の目的で使用する場合は、必要に応じ別途同意を対象者から取得する）。なお、試験の結果を公表する際も被験者を特定できる情報は使用しない。

- - 1. 安全性・不利益に対する配慮

本試験参加中の被験者になんらかの有害事象が発現した場合、担当医師は速やかに必要な対応（検査、治療、試験中止など）を行い、被験者の安全の確保に努める。

肝生検あるいは肝切除術は、さまざまなリスクを伴うが、本試験は3.1に示すように本試験への参加の有無に関わらず肝生検あるいは肝切除術が、日常診療の一環として必要と判断される患者を対象としている。

本試験への参加により追加される検査のうち、血液検査およびRTE、SWM、減衰計測、FibroScan、VTQ、SWE、SwSm、SWE_GEなどの超音波を用いた検査、あるいは、MR Touchは、危険性や不利益はないと考えられる。

- 1. 独立データモニタリング委員会および近畿大学医学部倫理委員会の承認

本試験の実施については、独立データモニタリング委員会および近畿大学医学部倫理委員会での承認を受ける。

1. **品質管理・品質保証**
   1. モニタリング
      1. 目的

試験が安全に、かつ試験実施計画書に従って実施されているか、データが正確に収集されているかを確認する。

- - 1. 中央モニタリング（in-house monitoring）

研究調査管理担当者に収集された症例報告書の記載内容、RTE、肝生検結果を対象とし、電子化されたデータの処理結果を参考として、データセンターが中央モニタリングを行う。なお、施設訪問モニタリングは予定していない。データセンターが作成する定期モニタリングレポートは、研究代表者および独立データモニタリング委員会に提出され検討される。

- - 1. 項目

1. 適格性
2. 症例集積状況、特に中止
3. 重篤な有害事象とその報告状況
4. その他
5. **記録の保存**

試験責任医師は、試験の実施等に関わる以下の文書を保管する。保管期間は、研究成果の発表後5年を経過した日までとする。

1. 申請書類の控え
2. 病院長からの通知文書
3. 各種申請書・報告書の控え
4. 被検者識別コードリスト
5. 同意書、被験者の同意に関する記録
6. 症例報告書等作成のための基礎データ（検査データ等）
7. **個人情報の管理と匿名化の方法**

病理標本および超音波検査データは、登録完了時に与えられる症例登録番号にて管理される。具体的には症例登録時に検体に対応する個人識別情報は英数字に変換し、以後、検体は符号化された連結可能匿名化番号のみで取り扱い、解析に用いる。

症例・符号対照表は、データセンターにより厳重に保管するものとし、測定機関の担当者に対して個人を特定するような臨床情報は一切伝えない。

1. **研究成果の発表**
   - 研究開始時点までにclinical trial registrationに登録（www.clinicaltrials.gov）し最終解析終了後に英文誌に投稿する。プロトコールで規定された最終解析までは、独立データモニタリング委員会の承認を得た場合を除いて発表は行わない。
   - ただし、研究代表者または研究事務局は、研究のエンドポイントの解析結果を含まない、研究の紹介目的の学会・論文（総説）発表は研究代表者およびSteering Committeeの了承を経て行うことができる。
   - 原則として、研究結果の主たる公表論文の著者は筆頭を研究事務局とし、以下、研究代表者、RTE、SWM及び減衰計測データ解析担当者、独立病理読影委員、統計解析責任者（公表のための解析を行った時点での担当者1名）の順とするが、研究代表者と協議して決定することとする。それ以下は、論文の投稿規定による制限に従って、登録数の多い順に施設研究責任者を施設毎に選び共著者とする。
   - 複数の異なった論文を書く場合、その筆頭者はSteering Committeeで協議し登録症例数の多い施設を重視する。
   - すべての共著者は投稿前に論文内容をreviewし、発表内容に合意した者のみとする。内容に関して議論にても合意が得られない場合、研究代表者はSteering Committee の了承の上でその研究者を共著者に含めないことができる。
   - 学会発表は複数回に及ぶ可能性があるため、研究事務局、研究代表者、登録の多い施設の研究責任者または研究実施施設医師の中から、持ち回りで発表を行うこととする。発表者は研究代表者が研究組織の代表者グループの了承を得て決定する。ただし、学会発表に際しては、発表準備および発表内容について研究事務局が責任を持ち、原則としてデータセンターとの連絡は研究事務局が行う。研究事務局以外の発表者が、研究事務局とデータセンターの了承なく、直接データセンターから集計・解析結果を受け取ることはできない。
2. **臨床試験登録**

本試験は、

既にClinicalTrial.gov ([www.clinicaltrials.gov](http://www.clinicaltrials.gov/))あるいはUMIN臨床試験登録システム（UMIN Clinical Trials Registry (UMIN-CTR)）に、登録予定である。

1. **利益相反（conflict of interest）と研究資金源**

本試験の計画、実施、発表に影響する可能性のある利益相反（conflict of interest）は想定していない。利益相反（conflict of interest）とは、研究成果に影響する様な利害関係を指し、金銭および個人の関係を含む。

本試験は、近畿大学医学部消化器内科研究費を補充し研究を実施する。

本試験の計画、実施、発表に関する意志決定は、研究代表者が行う。

1. **試験参加者の費用と健康被害の対応**
   1. 試験参加者の費用

本試験で採用する検査の内、RTE、SWM、減衰計測、VTQ、SWE、SwSm、SWE_GE、MR Touchは保険診療が認められていないため各試験実施施設の負担にて行う。一方、血液検査および肝生検あるいは肝切除術、FibroScanはいずれも保険適応承認が得られ、日常診療の範囲で行われていることから、RTE、SWM、減衰計測、VTQ、SWE、SwSm、SWE_GE、MR Touch以外の検査、入院費等を含む診療費はすべて患者の保険および自己負担により支払われる。

- 1. 健康被害の対応

本試験で健康被害が生じた場合は適切な治療を行い、その費用は通常の診療と同様に患者の保険および自己負担により支払われる。担当医師は、賠償責任に備え本試験開始前に賠償責任保険に加入する。

1. **試験実施計画の遵守、変更**
   1. 試験の終了、中止、中断
      1. 試験の終了

すべての施設において、試験実施期間終了（最終の患者登録から3ヶ月）時までの追跡が終了した時点を本試験終了とする。各施設での試験終了時には、試験責任医師は、速やかに試験終了報告書を病院長ならびに研究代表者に提出する。

- - 1. 試験の中止、中断

独立データモニタリング委員会は、必要に応じ試験の継続の妥当性を検討する。継続が適切でないと判断した場合には、同委員会は、本研究組織に試験の中止あるいは中断を勧告する。本研究組織が勧告に従い試験の中止を決定した場合には、研究代表者は可及的速やかに中止とその理由、試験中の参加者に対する対応方法を試験責任医師に伝達する。試験責任医師は経緯を倫理審査委員会（またはIRB）に報告し、研究代表者と倫理審査委員会（IRB）の指示に従い、試験中の参加者に対して適切な対応をとる。

試験責任医師は、以下の事項に該当する場合は試験実施継続の可否を検討する。

1. 試験治療の安全性、有効性に関する重大な情報が得られたとき。
2. 被験者のリクルートが困難で予定症例を達成することが到底困難であると判断されたとき。
3. 予定症例数または予定する試験実施期間に達する前に、（中間解析等により）試験の目的が達成されたとき。
4. 倫理審査委員会（IRB）により、実施計画等の変更の指示があり、これを受入れることが困難と判断されたとき。

試験責任医師は、倫理審査委員会（IRB）により中止の勧告あるいは指示があった場合は試験を中止する。試験の中止または中断を決定した時は、試験責任医師は速やかに病院長（あるいは各試験実施施設の長）にその理由とともに文書で報告する。

- 1. 試験実施計画書の遵守

本試験を行う研究者は、参加者の安全と人権を損なわない限りにおいて本試験実施計画書を遵守する。

- 1. 試験実施計画書からの逸脱

1. 担当医師は、研究代表者の事前の合意および倫理審査委員会の事前の審査に基づく病院長の承認を得る前に、試験実施計画書からの逸脱あるいは変更を行ってはならない。
2. 担当医師は、緊急回避等のやむを得ない理由により、研究代表者との事前の合意および倫理審査委員会の事前の承認を得る前に、試験実施計画書からの逸脱あるいは変更を行うことができる。その際には、担当医師は、逸脱または変更の内容および理由ならびに試験実施計画書等の改訂が必要であればその案を速やかに、研究代表者および倫理審査委員会に提出し、研究代表者、倫理審査委員会および病院長の承認を得るものとする。
3. 担当医師は、試験実施計画書からの逸脱があった場合は、逸脱事項をその理由とともにすべて記録し、試験責任医師は、各施設で定めた所定の様式により病院長および試験の代表者に報告しなければならない。試験責任医師は、これらの写しを保存しなければならない。
   1. 試験実施計画書の変更

試験実施計画書の変更並びに説明文書・同意書の変更あるいは改訂を行う場合は予め各施設の倫理審査委員会の承認を必要とする。また、承認を得るために本実施要項の変更を求められた場合、試験責任医師は、研究代表者との合意の上、当該施設での試験実施計画書を変更することができる。

- - 1. 試験実施計画書の変更の区分

中央倫理審査委員会（近畿大学医学部倫理委員会）の承認後の試験実施計画書の変更を改正・改訂の2種類に分けて取り扱う。定義と取り扱いは下記の通り。

1. 改正（Amendment）

試験の参加者の危険（risk）を増大させる可能性のある、または試験の主要評価項目に関連する試験実施計画書の部分的変更。
独立データモニタリング委員会および倫理審査委員会の審査承認を要する。承認を受けた場合は、カバーページに独立データモニタリング委員会の承認日を記載する。

1. 改訂（Revision）

試験の参加者の危険を増大させる可能性がなく、かつ試験の主要評価項目にも関連しない試験実施計画書の変更。
独立データモニタリング委員会の審査は不要だが、研究代表者の承認と独立データモニタリング委員会への報告を要する。倫理審査委員会の審査承認については各施設の取り決めに従う。承認を受けた場合は、カバーページに研究代表者の承認日を記載する。

- - 1. 試験実施計画書の改正／改訂時の施設倫理委員会承認

試験中に独立データモニタリング委員会の承認を得て本試験実施計画書または参加者への説明文書・同意書の改正がなされた場合は、改正された試験実施計画書および説明文書が倫理審査委員会（IRB）で承認されなければならない。

内容変更が改正ではなく改訂の場合に、倫理審査委員会の審査承認を要するか否かは各施設の取り決めに従う。

改正に対する倫理審査委員会承認が得られた場合、各施設の試験責任医師は倫理審査委員会承認文書のコピーをデータセンターへ送付する。倫理審査委員会承認文書原本は各施設が保管、コピーはデータセンターが保管する。

1. **研究組織**

本試験は研究組織が実施する自主臨床試験である。以下に本試験の関連組織について示す。

- 1. 研究代表者

工藤　正俊（近畿大学医学部　消化器内科）

- 臨床試験の総括
- 臨床試験実施体制の整備
- 試験実施計画書の決定
- 緊急時の対応
  1. 研究事務局

矢田　典久（近畿大学医学部　消化器内科）

〒589-8511　大阪府大阪狭山市大野東377-2

TEL : 072-366-0221（内線 3525）、FAX : 072-368-2880

E-mail : yada@med.kindai.ac.jp

- 1. Steering Committee（運営委員会）（順不同、敬称略）

工藤　正俊（近畿大学　消化器内科）

矢田　典久（近畿大学　消化器内科）

櫻井　俊治（近畿大学　消化器内科）

- 試験実施計画書・説明文書・同意書案の策定、変更の必要性の検討、改訂案の策定研究代表者への提示

- 1. RTE、SWMならびに減衰計測データ解析担当者

日立アロカメディカル㈱　第2メディカルシステム技術本部 第1技術開発部 開発2課

〒185-0014　東京都国分寺市東恋ヶ窪3-1-1

TEL: 042-329-4519(代表)

E-mail : tono4143@hitachi-aloka.co.jp

- 1. 独立病理読影委員（順不同、敬称略）

鹿毛　政義 (久留米大学 病理学)

坂元　亨宇（慶應義塾大学 医学研究科病理系専攻病理学）

中島 收 （久留米大学 医学部病理学）

- 病理学的解析
  1. 統計解析専門家（敬称略）

吉村　健一（京都大学医学部附属病院 探索医療センター）

- 研究デザインに関する統計的考察
- 統計解析手法の決定
- データ集計・統計解析・公表に関する統計的アドバイス
  1. 独立データモニタリング委員会（効果安全評価委員会）（順不同、敬称略）

委員長　 熊田　卓（大垣市民病院　消化器科）

委員 田中　正俊（ヨコクラ病院　消化器内科）

委員 岡　博子（ほうせんか病院）

- 円滑かつ適正な試験実施のためのモニタリング
- 中間解析の実施
- 中間解析の実施に基づくプロトコール改訂の必要性，試験継続の妥当性についての検討と研究代表者への提言
  1. データセンター

担当：弓削 公子

TEL : 072-366-0221（内線6458）、FAX : 072-367-1903

E-mail : kin-live@med.kindai.ac.jp

1. **試験実施施設**

本試験に参加する施設の条件は、試験を実施する診療科で肝生検に精通し、かつ日立アロカメディカル（株）HI VISION Ascendusを有し、RTE、SWM及び減衰計測が施行可能であること。

- 1. 試験実施施設（敬称略）
     - 1. 近畿大学医学部　消化器内科　工藤 正俊
       2. 武蔵野赤十字病院　消化器科　泉 並木
       3. 愛媛大学　消化器・内分泌・代謝内科学　日浅 陽一

1. **参考文献**

1. Imbert-Bismut, F., et al., *Biochemical markers of liver fibrosis in patients with hepatitis C virus infection: a prospective study.* Lancet, 2001. **357**(9262): p. 1069-75.

2. Gressner, A.M., C.F. Gao, and O.A. Gressner, *Non-invasive biomarkers for monitoring the fibrogenic process in liver: a short survey.* World J Gastroenterol, 2009. **15**(20): p. 2433-40.

3. Castera, L., *Transient elastography and other noninvasive tests to assess hepatic fibrosis in patients with viral hepatitis.* J Viral Hepat, 2009. **16**(5): p. 300-14.

4. Castera, L., et al., *Prospective comparison of transient elastography, Fibrotest, APRI, and liver biopsy for the assessment of fibrosis in chronic hepatitis C.* Gastroenterology, 2005. **128**(2): p. 343-50.

5. Foucher, J., et al., *Diagnosis of cirrhosis by transient elastography (FibroScan): a prospective study.* Gut, 2006. **55**(3): p. 403-8.

6. Ganne-Carrie, N., et al., *Accuracy of liver stiffness measurement for the diagnosis of cirrhosis in patients with chronic liver diseases.* Hepatology, 2006. **44**(6): p. 1511-1517.

7. Sagir, A., et al., *Transient elastography is unreliable for detection of cirrhosis in patients with acute liver damage.* Hepatology, 2008. **47**(2): p. 592-5.

8. Tatsumi, C., et al., *Noninvasive evaluation of hepatic fibrosis using serum fibrotic markers, transient elastography (FibroScan) and real-time tissue elastography.* Intervirology, 2008. **51 Suppl 1**: p. 27-33.

9. Masuzaki, R., et al., *Risk assessment of hepatocellular carcinoma in chronic hepatitis C patients by transient elastography.* J Clin Gastroenterol, 2008. **42**(7): p. 839-43.

10. Millonig, G., et al., *Extrahepatic cholestasis increases liver stiffness (FibroScan) irrespective of fibrosis.* Hepatology, 2008. **48**(5): p. 1718-23.

11. Nguyen-Khac, E., et al., *The non-invasive diagnosis of cirrhosis using the Fibroscan must be performed with cause-specific stiffness cut-offs.* Gut, 2008. **57**(11): p. 1630; author reply 1630-1.

12. Lucidarme, D., et al., *Factors of accuracy of transient elastography (fibroscan) for the diagnosis of liver fibrosis in chronic hepatitis C.* Hepatology, 2009. **49**(4): p. 1083-9.

13. Wang, J.H., et al., *FibroScan and ultrasonography in the prediction of hepatic fibrosis in patients with chronic viral hepatitis.* J Gastroenterol, 2009. **44**(5): p. 439-46.

14. Itoh, A., et al., *Breast disease: clinical application of US elastography for diagnosis.* Radilogy, 2006. **239**:p.341-50.

15. Tsutsumi, M., et al., *Real-Time Balloon Inflation Elastography for Prostate Cancer Detection and Initial Evaluation of Clinicopathologic Analysis.* AJR, 2010. **194**(6):p.W471-6.

16. Fukunari, N., et al., *More Accurate and Sensitive Diagnosis for Thyroid Tumors with Elastography - Detection and Differential Diagnosis of Thyroid Cancers.*  MEDIX Supplement, 2007: p. 16-19.

17. Friedrich-Rust, M., et al., *Real-time elastography for noninvasive assessment of liver fibrosis in chronic viral hepatitis.* AJR Am J Roentgenol, 2007. **188**(3): p. 758-64.

18. Fujimoto, K., et al., *Non-invasive evaluation of Hepatic Fibrosis in patients with Chronic Hepatitis C using Elastography.* MEDIX Supplement, 2007: p. 24-27.

19. Friedrich-Rust, M., et al., *Real-time tissue elastography versus FibroScan for noninvasive assessment of liver fibrosis in chronic liver disease.* Ultraschall Med, 2009. **30**(5): p. 478-84.

20. Arena, U., et al., *Acute viral hepatitis increases liver stiffness values measured by transient elastography.* Hepatology, 2008. **47**:p.380-84.

21. Cobbold, J.F., et al., *Transient elastography in acute hepatitis: all that's stiff is not fibrosis.* Hepatology, 2008. **47**:p.370-72.

22. Colli, G., et al., *Decompensated chronic heart failure: increased liver stiffness measured by means of transient elastography.* Radiology, 2010. **257**:p.872-78.

23. Yada, N., et al., *Assessment of Liver Fibrosis with Real-time Tissue Elastography in Chronic Viral Hepatitis.* Oncology, 2013. **84 (suppl 1)**: p. 13-20.

24. Maklad, N.F., et al., *Attenuation of ultrasound in normal liver and diffuse liver disease in vivo.* Ultrason Imaging 1984;**6**:p.117-25.

25. Taylor, K.J., et al., *Quantitative US attenuation in normal liver and in patients with diffuse liver disease:Importance of fat.* Radiology 1986;**160**:p.65-71.

26. Wilson, L.S., et al., *Evaluation of ultrasonic attenuation in diffuse diseases of spleen and liver..* Ultrason Imaging 1987;**9**:p.236-47.

27. Garra, B.S., et al., *Quantitative estimation of liver attenuation and echogenicity: Normal state versus diffuse liver disease..* Radiology 1987;**162**:p.61-67.

28. Sasso, M., et al., *Controlled attenuation parameter(CAP): a novel VCTETM guided ultrasonic attenuation measurement for the evaluation of hepatic steatosis: preliminary study and validation in cohort of patients with chronic liver disease from various causes.* Ultrasound Med Biol. 2010;**36**(11):p.1825-35.
