# Supplementary material for: New diagnostic technique to evaluate hepatic steatosis using the attenuation coefficient on ultrasound B mode
Source: PLoS One. 2019 Aug 27;14(8):e0221548. doi: 10.1371/journal.pone.0221548 (PMC6711590; doi:10.1371/journal.pone.0221548)
Supplement: S2 Protocol — (DOCX) [file pone.0221548.s005.docx]

The usefulness of noninvasive liver function comprehensive evaluation technique by ultrasound

- Research plan -

・**Request for confidentiality**

・This plan is the intellectual property of this research steering committee. Therefore, we ask that you do not disclose or disclose information on this research plan to third parties without prior permission from the document from this study group.

First edition made on June 29, 2015

Contents

Page

0. Outline of the test 3

1. Purpose 5
2. Background 5
3. Target 7
4. Examination plan 8
5. Survey and inspection 10
6. Evaluation and report of adverse events 15
7. Data collection 17
8. End point 18
9. Statistics 18
10. Ethics 20
11. Quality control and quality assurance 21
12. Save record 23
13. Management of personal information and methods of anonymization 23
14. Presentation of research results 23
15. Clinical trial registration 24
16. Conflicts of Interest and Research Funding Sources 25
17. Response to expense of test participants and health damage 25
18. Compliance with the test implementation plan, change 25
19. Research organization 28
20. Examination facility 30
21. References 31

**Outline of the test**

0.1. Examination plan

Study on evaluation of liver function by elastography and attenuation measurement for liver disease patients who undergo liver biopsy or liver resection.

Liver disease patient who undergoing liver biopsy or liver resection

Perform the following test within 4 months before or after liver biopsy or hepatectomy. Blood test, Real-time Tissue Elastography (RTE), Attenuation Measurement, FibroScan, Virtual Touch Quantification (VTQ), ShearWave Elastography (SWE), Shear Wave with Smart Maps (SwSm), Shear Wave Elastography (SWE_GE), MR Touch

0.2. Purpose

We perform Real-time Tissue Elastography (RTE), Shear Wave Measurement (SWM) and attenuation measurement on liver disease patients who undergo liver biopsy or liver resection. We develop an algorithm to estimate indices of liver fibrosis, liver inflammation, liver steatosis by these modality.

At the same time, these indicators and liver tissue diagnosis, blood test, or other elastography (FibroScan, Virtual Touch Quantification (VTQ), ShearWave Elastography (SWE), Shear wave with Smart maps (SwSm), Shear Wave Elastography (SWE_GE) MR Touch) are compared. Investigate whether we can evaluate the general condition of the liver non-invasively such as liver fibrosis, liver inflammation, and liver steatosis.

0.3. Content of evaluation

0.3.1. Primary endpoint

- Develop algorithms to calculate indices of hepatic fibrosis, inflammation, fatty liver using RTE, SWM, and attenuation measurement.

0.3.2 Secondary endpoints

- Relationship between indicators calculated using RTE, SWM, attenuation measurement and blood test results.
- Correlation between indicators calculated using RTE, SWM, attenuation measurement and pathological diagnosis.
- Correlation between indicators calculated using RTE, SWM, attenuation measurement and other elastography.

0.4. Target

0.4.1. Target case

Applicable to those who fall under ① or ②.

① Liver disease patient undergoing liver biopsy or liver resection.

② Healthy volunteers.

Serologically or diagnostically diagnosing liver disease can be denied as a healthy individual.

0.4.2. Selection criteria

Unless otherwise specified, the results within six months prior to registration shall be used for inspections and findings.

1. Age / Sex: Male and female over 20 years old.
2. After receiving sufficient explanation for participation in this study, patients who obtained written consent by the patient's free will with sufficient understanding.

[Rationale for setting selection criteria]

(1) Targeted aged 20 and over who can obtain consent from the principal.

(2) Based on the Declaration of Helsinki.

0.4.3. Exclusion Criteria

(1) Patients who cannot measure elastography ROI (Region of Interest) from tumor.

(2) Pregnant women and patients who may be pregnant or breast-feeding patients.

(3) Other patients who are deemed inappropriate by the research doctor.

[Rationale for Setting Exclusion Criteria]

(1) We excluded cases where adequate measurement of nonelastograph elastography was not possible.

(2) - (3) Safety was taken into consideration.

0.5. Target number of subjects and scheduled expected duration.

- Target sample size

Total of 450 cases (400 cases of biopsy or surgical cases, 50 healthy volunteers)

- Registration period

From the approval of the Ethics Committee until March 31, 2017. However, shorten or extend the period as necessary.

- Examination period

From the approval of the ethics committee until the last 3 months after registration of the patient. However, shorten or extend the period as necessary.

0.6. Contact address

Research Bureau

Norihisa Yada

Department of Gastroenterology and Hepatology, Faculty of Medicine, Kindai University, Osaka, Japan, 589-8511.

TEL: +81-72-366-0221

E-mail: yada@med.kindai.ac.jp

1. Purpose

We perform Real-time Tissue Elastography (RTE), Shear Wave Measurement (SWM) and attenuation measurement on liver disease patients who undergo liver biopsy or liver resection. We develop an algorithm to estimate indices of liver fibrosis, liver inflammation, liver steatosis by these modality.

At the same time, these indicators and liver tissue diagnosis, blood test, or other elastography (FibroScan, Virtual Touch Quantification (VTQ), ShearWave Elastography (SWE), Shear wave with Smart maps (SwSm), Shear Wave Elastography (SWE_GE) MR Touch) are compared. Investigate whether we can evaluate the general condition of the liver non-invasively such as liver fibrosis, liver inflammation, and liver steatosis.

2. Background

2.1. Background

Chronic liver disease caused by HBV or HCV has a higher risk of carcinogenesis as the disease progresses, and increases the risk of portal hypertrophy such as gastroesophageal varices and liver failure. Liver cirrhosis is also known as one of the therapeutic resistant host factors for interferon. In order to treat these chronic liver diseases, it is important to evaluate and understand liver fibrosis, inflammation, and fatty liver.

Liver biopsies are important for definitive diagnosis of liver fibrosis, inflammation, and steatosis, but they are also invasive and may cause sampling errors.

In fibrosis, various serum markers have been reported as noninvasive indices [1-3]. In recent years, various devices for measuring liver hardness using ultrasonic technology have been developed, and many reports that good correlation between liver hardness and degree of fibrosis has been obtained. In particular, many reports on FibroScan, which calculates the shear modulus of liver tissue by detecting the transmission rate of vibration oscillated from the probe, have obtained good results [4-13]. On the other hand, RTE can visualize tissue strain distribution by manual compression and vibration in real time, and its effectiveness in breast tumor, prostate tumor, thyroid tumor has been reported [14-16]. Likewise, in the liver a small number of possibilities are useful for measurement of fibrosis degree from distorted images due to heartbeat [8,17,18]. There are reports that it is more useful than FibroScan and serum markers [19, 20]. However, an appropriate evaluation method has not been determined. In addition, the propagation speed of the shear wave generated when the strain (shear) of the tissue generated by the technique of Acoustic Radiation Force Impulse (ARFI) which micro displacement of the soft tissue using the acoustic radiation pressure returns to the original Many reports that Virtually Touch Quantification (hereinafter referred to as VTQ), ShearWave Elastography (hereinafter SWE), Shear wave with Smart maps (hereinafter SwSm), Shear Wave Elastography (hereinafter SWE_GE) are useful as a tool for measuring liver stiffness. Furthermore, MR Elastography, which measures liver stiffness using MRI, has also been developed recently, and MR Touch has started to be clinically applied.

Liver stiffness measured by Shear wave imaging such as FibroScan, VTQ, SWE, SwSm, SWE_GE is affected by inflammation, jaundice, congestion and the like besides fibrosis [10, 20-22], whereas RTE influences inflammation There are reports that it can evaluate fibrosis without receiving it [23]. Therefore, by simultaneously measuring both, there is a possibility that the extent of inflammation and fibrosis of the liver can be evaluated separately.

On the other hand, steatosis of the liver, a method to obtain it from the attenuation coefficient of ultrasonic wave has been reported in the 1980 's [24-27], FibroScan measures liver hardness and simultaneously determines attenuation coefficient of ultrasonic wave Controlled Attenuation Parameter (CAP) has been installed and its usefulness has been reported [28].

In this issue, SWM, which is Shear wave elastography, and attenuation measurement function have been developed from Hitachi Aloka Medical, Inc. and it has become possible to measure on the same equipment as RTE which is conventional Strain elastography.

Therefore, for liver disease cases subject to liver biopsy or hepatectomy, indexes of hepatic fibrosis, inflammation, fatty liver are calculated using the results of pathological diagnosis as teacher data, using the results of RTE, SWM and attenuation measurement Consider an algorithm to do. In addition, blood collection test, pathological diagnosis, FibroScan, VTQ, SWE, SwSm, SWE_GE and MR Touch are performed, their correlation is evaluated, and its usefulness is examined.

3. Target

3.1. Target case

Applicable to those who fall under ① or ②.

① Liver disease patient undergoing liver biopsy or liver resection.

② Healthy volunteers.

Serologically or diagnostically diagnosing liver disease can be denied as a healthy individual.

3.2. Selection criteria

Unless otherwise specified, the results within six months prior to registration shall be used for inspections and findings.

1. Age / Sex: Male and female over 20 years old.
2. After receiving sufficient explanation for participation in this study, patients who obtained written consent by the patient's free will with sufficient understanding.

[Rationale for setting selection criteria]

(1) Targeted aged 20 and over who can obtain consent from the principal.

(2) Based on the Declaration of Helsinki.

3.3. Exclusion Criteria

(1) Patients who cannot measure elastography ROI (Region of Interest) from tumor.

(2) Pregnant women and patients who may be pregnant or breast-feeding patients.

(3) Other patients who are deemed inappropriate by the research doctor.

[Rationale for Setting Exclusion Criteria]

(1) We excluded cases where adequate measurement of nonelastograph elastography was not possible.

(2) - (3) Safety was taken into consideration.

Examination plan

4.1. Examination design

Multicenter cross-sectional test

4.1.1. Overall test outline

- Blood sampling, ultrasound elastography (RTE, SWM, FibroScan, VTQ, SWE, SwSm, SWE_GE), attenuation measurement, MR Touch are performed within 4 months before or after liver biopsy or liver resection.
- Blood is drawn on the same day as ultrasonic elastography and attenuation measurement enforcement.

4.1.2. Target number of subjects and scheduled expected duration.

- Target sample size

Total of 450 cases (400 cases of biopsy or surgical cases, 50 healthy volunteers)

- Registration period

From the approval of the Ethics Committee until March 31, 2017. However, shorten or extend the period as necessary.

- Examination period

From the approval of the ethics committee until the last 3 months after registration of the patient. However, shorten or extend the period as necessary.

4.2 Case registration

(1) The attending physician will explain this research to patients satisfying eligibility requirements and obtain consent.

(2) The doctor in charge will satisfy all selection criteria for patients agreeing to the registration, confirm that they do not fall under any exclusion criteria, complete all the necessary items in the "case registration card" and send it to the data center Fax the "case registration card".

(3) The data center confirms the contents and eligibility of the case registration form, confirms the contents of the case registration inquiries to the attending physician, confirms that all the criteria are satisfied, and accepts the registration.

(4) The data center faxes the result of qualified / ineligible to the FAX number of the attending physician described in the "case registration card". If qualified, issue "Registration Confirmation Form" containing case registration number. With FAX transmission of the registration confirmation letter, "registration completed" is set. If it is ineligible, FAX-sending an "ineligibility notice" to the attending physician.

(5) The doctor in charge stores the original of the case registration form and the registration confirmation. The data center stores as a copy of the case registration slip sent by FAX.

<Registration>

Data center

Officer: Kimiko Yuge

TEL: +81-72 - 366 - 0221

E-mail: kin-live@med.kindai.ac.jp

<Contact address>

Research Bureau

Norihisa Yada

Department of Gastroenterology and Hepatology, Faculty of Medicine, Kindai University, Osaka, Japan, 589-8511.

TEL: +81-72-366-0221

E-mail: yada@med.kindai.ac.jp

4.2.1. Precautions for Registration

1) The registration date shall be the date on which a series of registration procedures has been completed and shall be stated in the registration confirmation. It is not "registered" at the time of faxing the case registration form to the data center.

2) Registered patients are not canceled (deleted from the database) unless there is a withdrawal of agreement including rejection of data research use. In the case of duplicate registration, in any case the first registration information (case registration number) is adopted.

3) If mis-registration or duplicate registration is found out, contact the data center promptly.

4.3. Definition of study completion

"Completion of study" is defined as the case where follow-up is completed until the death or observation period after the test treatment. The case where the follow-up observation was stopped before the death or the end of the observation period shall be referred to as "the study is canceled".

4.4 Cancellation before study Inspection

If the doctor in charge is found to be ineligible at the time of registration after registration of the patient, if there is an offer to stop examination examination from the patient, and the doctor in charge judges that the examination treatment is inappropriate, Stop test examination. If you terminate the examination before starting the examination, record the reason and details of discontinuation in the medical record and case report.

4.5. Combination therapy

4.5.1 Concomitant Prohibited Therapy

Do not set prohibited drugs.

4.6. Follow-up observation after inspection

After the examination, observe whether there was no side effect associated with the examination for 30 days.

5. Survey and examination

5.1.1. Patient background

- Birth date, age, sex, height, weight, waist circumference, thickness from the body surface to the hepatic parenchyma

As with the metabolic syndrome diagnosis, the abdominal girth measurement method is measured in the standing position, light exhalation, around the umbilicus, but when fat accumulation is pronounced and the navel deviates downward, the rib lower border and the anterior superior Measure at the height of the midpoint of the iliac spine.

- PS (performance status).
- For hepatectomy purpose (primary liver cancer, metastatic liver cancer, others).
- Liver etiology (hepatitis C, hepatitis B, alcoholic liver disease, nonalcoholic fatty liver disease etc.).
- Alcohol Intake Period.
- Whether the patient is receiving antiviral treatment. Treatment content.
- Whether the patient has pleural effusion or ascites.
- Whether the patient has arrhythmia.
- Whether the patient has heart disease.
- Other medical history or complications.

5.2. Survey after registration

- Blood test, ultrasound elastography (RTE, SWM, FibroScan, VTQ, SWE, SwSm, SWE_GE), attenuation measurement, MR Touch are performed within 4 months before or after liver biopsy or hepatectomy.
- Blood is drawn on the same day as ultrasonic elastography and attenuation measurement enforcement.

5.2.1 Blood test

5.2.1. Blood test

Of the following tests, those underlined ( ) do blood test on an empty stomach.

Biochemistry: AST, ALT, γGTP, ALP, total bilirubin, direct bilirubin, total protein, albumin, protein fraction, cholinesterase, total cholesterol, LDL cholesterol, HDL cholesterol, triglyceride, BUN, Cr, plasma glucose, HbA1c, insulin, ferritin, serum Fe, TIBC, UIBC.

Blood cell: white blood cell count, red blood cell count, hemoglobin, hematocrit, number of platelets.

Coagulation: prothrombin time (%, INR).

Fibrotic marker: hyaluronic acid, type IV collagen 7S, procollagen III peptide (P-Ⅲ-P), M2BPGi.

5.2.2. Liver pathology

- Liver biopsy or liver resection pathology specimens are stained with ① HE staining and ② masson trichrome staining.
- Make the slide virtual slide and send it to independent pathology interpretation committee member.
- Independent pathology interpretation committee evaluates fibrosis in accordance with new inuyama classification (complies with METAVIR classification).

5.2.3. Real-time Tissue Elastography® (RTE)

- Equipment used: Ascendus (Hitachi Aloka Medical, Inc. Tokyo, Japan).
- Transducer: Convex probe EUP - C 715
- Details of the measurement method are shown in the separate sheet (RTE imaging method).
- Data will be anonymized and sent to Hitachi Aloka Medical, Inc., and analyzed with Hitachi Aloka Medical, Inc. blinded the patient background.

5.2.4. Shear Wave Measurement (SWM)

- Equipment used: Ascendus (Hitachi Aloka Medical, Inc. Tokyo, Japan).
- Transducer: Convex probe EUP - C 715
- Details of the measurement method are shown in a separate sheet (SWM imaging method).
- Data will be anonymized and sent to Hitachi Aloka Medical, Inc., and analyzed with Hitachi Aloka Medical, Inc. blinded the patient background.

5.2.5. Attenuation measurement

- Equipment used: Ascendus (Hitachi Aloka Medical, Inc. Tokyo, Japan).
- Transducer: Convex probe EUP - C 715
- Details of the measurement method are shown in a separate sheet (SWM imaging method).
- Data will be anonymized and sent to Hitachi Aloka Medical, Inc., and analyzed with Hitachi Aloka Medical, Inc. blinded the patient background.

5.2.6. Vibration-Controlled Transient Elastography (VCTE)

- For facilities owned by FibroScan, evaluation is also performed with Vibration-Controlled Transient Elastography within 4 months before and after liver fibrosis evaluation by liver biopsy, hepatectomy, RTE, or SWM.
- Transducer: M-probe, XL-probe
- Inspection 10 times at the right intercostal space, validity of 60% or more is recognized, and when the IQR is less than 1/3 of the measured value, the examination is judged as valid.

5.2.7. Controlled Attenuation Parameter (CAP)

- For institutions that own FibroScan, assessment by CAP is also conducted within 4 months before and after liver fat estimation by liver biopsy, hepatectomy, and attenuation measurement function.
- Transducer: M-probe, XL-probe
- Inspection 10 times at the right intercostal space, validity of 60% or more is recognized, and when the IQR is less than 1/3 of the measured value, the examination is judged as valid.

5.2.8. Virtual touch quantification (VTQ)

- For facilities that own ACUSON S2000, assessment with VTQ is also conducted within 4 months before and after liver fibrosis evaluation by liver biopsy, hepatectomy, RTE, or SWM.
- Used equipment: Mochida SIEMENS company ACUSON S2000
- Transducer: 4C1
- Expose the right lobe of the liver by manipulation of the right intercostal space, place the ROI 1 - 4 cm below the liver surface, check 5 times and calculate median.
- At the time of measurement, make the liver surface as horizontal as possible (parallel to the probe) with respect to the echo screen.

5.2.9. ShearWave Elastography (SWE)

- For institutions that own Aixplorer, also evaluate with SWE within 4 months before and after liver fibrosis evaluation by liver biopsy, hepatectomy, RTE, or SWM.
- Equipment used: Konica Minolta Aixplorer
- Transducer: SC 6 - 1
- Right liver intercostal operation to depict the right lobe of the liver, place ROI several cm below the liver surface so as not to be affected by multiple reflections, check 5 times and calculate median.
- At the time of measurement, make the liver surface as horizontal as possible (parallel to the probe) with respect to the echo screen.

5.2.10. Shear wave with Smart maps (SwSm)

- For institutions that own Aplio 500 Platinum, also evaluate with SwSm within 4 months before and after liver biopsy, hepatectomy, liver fibrosis evaluation by RTE or SWM.
- Used equipment: Toshiba Aplio 500 Platinum
- Transducer: 375 BT
- Right liver intercostal operation to depict the right lobe of the liver, place ROI several cm below the liver surface so as not to be affected by multiple reflections, check 5 times and calculate median.
- At the time of measurement, make the liver surface as horizontal as possible (parallel to the probe) with respect to the echo screen.

5.2.11. MR Touch

- For institutions that own MR Touch, also evaluate with MR Touch within 4 months before and after liver fibrosis evaluation with liver biopsy, hepatectomy, RTE, or SWM.
- Equipment used: GE Healthcare Japan Co., Ltd. MR Touch compatible model

5.2.12. Shear Wave Elastography (SWE_GE)

- For institutions that own LOGIQ E 9, also evaluate with SWE_GE within 4 months before and after liver fibrosis evaluation by liver biopsy, hepatectomy, RTE, or SWM.
- Equipment used: GE LOGIQ E 9
- Transducer: C1-6-D or C1-6VN-D probe
- Right liver intercostal operation to depict the right lobe of the liver, place ROI several cm below the liver surface so as not to be affected by multiple reflections, check 5 times and calculate median.
- At the time of measurement, make the liver surface as horizontal as possible (parallel to the probe) with respect to the echo screen.

6. Evaluation and report of adverse events

Adverse events are defined as any undesirable symptoms and signs (including anomalies in laboratory test values) expressed in subjects, with or without a causal relationship to the study treatment. If an adverse event occurs, the attending physician promptly carries out the necessary response (inspection, treatment, discontinuation of examination, etc.) and strives to ensure the safety of the subject. We also evaluate and report on adverse events according to the procedures specified below.

6.1. Evaluation of adverse events

6.1.1. Adverse Events to be Evaluated

In this study, among serious adverse events that occurred within 30 days after the start of the study treatment since the start of the study treatment, those that are not denied the relation with the test examination shall be evaluated. However, worsening of the original disease is excluded.

6.1.2. Survey

The doctor in charge investigates the occurrence of the adverse event to be evaluated. Investigate the following items and fill in the medical record and case report.

We observed the event name (in principle diagnosis name), the date of onset, severity (severe, non-serious), the presence or absence of treatment, degree (mild, moderate, severe), course (disappearance, alleviation, invariance, deterioration Day), causal relationship with examination examination (related, maybe relevant, probably not related, unrelated) and reason for judgment.

6.1.3. Serious adverse events

A case that falls under any of the following is regarded as a serious adverse event.

(1) Things that lead to death.

(2) threatening life (if the patient is in danger of death when the event occurs).

(3) Requiring hospitalization for treatment or extension of hospitalization period.

(4) Persistent or noticeable failure and malfunction.

(5) Causes congenital anomalies.

(6) Other medically important conditions (so as not to immediately life threaten or lead to death or hospitalization, subjecting the patient to crisis or requiring treatment so as not to result in the above result Serious event).

6.1.4. Degree

Mild: Those that are not treated and can be continued to be tested or those that do not interfere with everyday life

Moderate: Although treatment is necessary, the extent to which the test can be continued or which interferes with daily life

Severe: The extent to which the test cannot be continued (excluding stoppage due to the subject's offer) or impossible daily life

6.1.5. Process

Fill in the judgment date in the case report and keep track of the progress until "disappearance" as much as possible.

(1) Disappearance: When symptoms disappear.

(2) Reduction: When symptoms are alleviated.

(3) invariant: unchanged symptoms.

(4) Worsening: symptoms worsened.

(5) Unknown.

6.1.6. Criteria for causality with test examination

Based on the following factors, it is judged in 5 stages (relevant, perhaps related, probably not related, no relevant) about the existence of reasonable possibility that the adverse event was considered to have been caused by the test examination.

Factors of reasonable possibility

(1) Time course: There is a reasonable time relationship between the test examination and the occurrence of an adverse event.

(2) Known treatment characteristic: It can be predicted from the known information of the relevant test examination or from the characteristics of the examination examination.

(3) Existence of other causes: In addition to other reasons, rational explanation can be made with other treatments, original diseases, underlying diseases, host factors, environmental factors and the like.

(4) Specific examination: Causal relationship is proved by specific examination.

6.2. Adverse event report

The doctor in charge of each facility promptly notifies the data center of the serious adverse event emergency report promptly when admitting the evaluation target adverse event (serious adverse event which is not denied the relation with the examination) FAX transmission is performed. The data center regularly reports on the occurrence of the adverse event to be evaluated to the research representative. The research representative shares information with the research group and the test execution facility about the occurrence of the adverse event to be evaluated.

6.2.1. Reporting to the Independent Data Monitoring Committee

(1) The research representative shall periodically report to the Independent Data Monitoring Committee on the adverse event to be evaluated reported from each facility, and at the same time, appropriately reflect the viewpoint of the research representative for the adverse event and the response to the adverse event Ask for examination on sex.

(2) The Independent Data Monitoring Committee reviews the content of the report and recommends to the research delegate in writing the future response including handling of cases and whether to continue the registration.

7. Data collection

7.1. Case report form (CRF)

7.1.1. Data submission

In this study, case registration forms and other case report data are submitted by FAX. The doctor in charge or the clinical research coordinator (CRC) etc. will submit data using FAX to the data center according to the progress of the examination, for all cases registered in this examination until the examination is completed. In the case where CRC or the like fills in or inputs, confirmation by the attending physician is obtained. The contents of the data to be submitted, the means of submission and the submission date are shown below.

7.1.2. Case report type and submission deadline

The types of CRF used in this study, the means and timing of sending and submitting are shown below.

| No. | Type | Means of sending to facilities, timing | Means of submission |
| --- | --- | --- | --- |
| 1 | Case registration card | - Mailing - Immediately after registering facilities | - FAX transmission - At the time of registration |
| 2 | Case registration card | - Mailing - Immediately after registering facilities | - FAX transmission - Within one month from each survey |
| 3 | Serious adverse event emergency report | - The doctor can also use the each-hospital format - Mail in advance to participating facilities | - FAX transmission - Immediately after knowing the occurrence of an emergency reporting adverse event |

7.1.3. Data management

In accordance with a separately specified data management plan (standard operating procedure and manual), the data center performs dunning of data not submitted, scrutiny and inquiries of the submitted data, data correction based on the inquiry result, database management. In addition, the data center prepares data for monitoring and data set for statistical analysis.

8. Endpoint

8.1. Endpoint definition

8.1.1. Primary endpoint

Develop algorithms to calculate indices of hepatic fibrosis, inflammation, fatty liver using RTE, SWM, and attenuation measurement

8.1.2. Secondary points (Secondary Evaluation Item)

- Relationship between indicators calculated using RTE, SWM, attenuation measurement and blood test results.
- Relationship between indicators calculated using RTE, SWM, attenuation measurement and pathological diagnosis.
- Correlation between indicators calculated using RTE, SWM, attenuation measurement and other elastography and attenuation measurements.

9. Statistical matters

9.1. Definition of analysis target group

The group to be analyzed used for periodic monitoring, intermediate analysis, and final analysis is defined as follows.

(1) All registration example: All patients registered in accordance with the registration procedure are "all registered examples".

(2) Ineligible Example: Among registered patients, patients who do not satisfy the registration criteria (the fact that they do not agree with each other later) are referred to as "ineligible cases".

(3) All qualified examples: The group excluding "incapacitated cases properly decided in the research group" from all registered examples shall be regarded as "all qualified examples".

9.2. Analysis of Primary endpoint

Regarding the association between pathological diagnosis and RTE / SWM / attenuation measurement, index of liver fibrosis, inflammation, fatty liver is calculated using multiple regression analysis and data mining etc. Also, sub-analysis is performed for each disease and examined.

9.3. Analysis of Secondary endpoints

Pearson correlation coefficients and so on are evaluated to evaluate the association between the indicators of hepatic fibrosis, inflammation and fattying by each elastography and attenuation measurement and serum markers, respectively.

Pearson correlation coefficients and so on are evaluated in order to evaluate the relevance between the indicators of hepatic fibrosis, inflammation and fattying by each elastography and attenuation measurement and the pathological diagnosis.

Pearson correlation coefficients and so on are evaluated to evaluate correlations between liver fibrosis, inflammation, fatty index and other elastography and attenuation measurements by each elastography and attenuation measurement, respectively.

Also, sub-analysis is performed for each disease and examined.

9.4. Rationale for setting the target number of subjects

In order to calculate indices of liver fibrosis, inflammation, fatty liver, 300 cases of liver biopsy or liver resection or 30 cases of healthy cases are required as teacher data. In addition, 100 cases of liver biopsy or hepatectomy cases and 20 cases of healthy cases are required to verify the calculated usefulness of the liver fibrosis, inflammation and fatty index. Since there are 250 cases of liver biopsy cases and 50 cases of hepatectomy cases at the institute, we can easily compare them with 400 cases of pathological diagnosis for a total of 3 institutions. Also, 20 healthy volunteers are planned to be imaged at each facility.

9.5. Exploratory analysis by accompanying research

In the case of newly subordinate research using the data of this study, we summarize the contents to be studied and submit it to this research leader. The research leader will examine the content and implementation method of the incidental research that was proposed together with the proposer.

10. Ethics

All researchers involved in this examination received the Helsinki Declaration and the Ministry of Health, Labor and Welfare's "Ethics Guidelines on Clinical Studies"(http://www.mhlw.go.jp/stf/seisakunitsuite/bunya/hokabunya/kenkyujigyou/i--kenkyu/index.html) and carry out this test.

10.1. Informed consent

10.1.1. Acquisition of explanation and consent to the patient

Before the patient participates in the exam, the attending physician hands out the explanatory document approved by the institutional ethics review committee (or the Institutional Review Board, IRB) to the patient orally and fully. After explaining the test, give enough time to judge as an opportunity to ask a question, ask the patient to understand the contents of the exam well, ask the participation in the exam, depending on the individual's willingness to do so Consent shall be obtained with consent form.

In addition to the physician in charge who explained the explanation, in the consent form, when the test cooperator makes a supplementary explanation, the relevant test cooperator has written, signed or signed the respective date, respectively, and the subject Please fill in the consent date and stamp or sign and sign. A copy of the consent form is provided to the subjects, and the original is preserved in the facility.

10.1.2. Continuous participation in the examination When information possibly affecting the intention of the subject is obtained

(1) If the subject physician got information that may influence the intention of the subject concerning continued participation in the examination while participating in the exam, the attending physician immediately described the information Provide explanatory materials and explain to the subjects the following on the basis.

① About this information

② It is free to continue participation in this exam

(2) In the explanatory document / consent form, the doctor in charge who made the explanation fills in the date of explanation and stamps or signs it, and the examinee fills in the date of receipt of the information and stamps or signs the above name. In addition, if a test cooperator makes a supplementary explanation, the relevant test cooperator also fills in the date and stamps or signs it. A copy of the material shall be provided to the subjects. The attending physician confirms the intention of the subject whether or not to continue participating in this examination, and fills in the intention confirmation date and the confirmation result on the original document of the explanation document / consent form and saves it.

(3) After revising the explanatory document / consent form and obtaining approval from the ethics review committee, the attending physician will explain it again using the revised explanatory document and consent form to the subject, and the participating in this examination For consecutive purposes we obtain written consent from free volunteers from subjects. On that occasion, the doctor in charge fills in the explanation date in the consent form, and stamps or signs it, and the subject also signs and signs or signs after entering the date of consent. A copy of the consent form will be provided to the subject and the original will be stored at the facility.

10.2. Consideration concerning human rights, safety and disadvantage of subjects

10.2.1. Consideration for human rights (protection of privacy)

When dealing with raw data related to the test implementation and agreement form etc, pay close attention to the secrecy protection of the subjects. Also, even in preparation and handling of case report to be submitted outside the hospital, the subject is identified by "case registration number" issued at the time of registration assignment and "subject identification code" set within the facility, and regarding the secret protection consider. Data of subjects obtained in this study will not be used for purposes other than the purpose of this study (if used for purposes other than this research, separate consent will be obtained from the subject as necessary). In addition, when publishing the results of the examination, do not use information that can identify subjects.

10.2.2. Consideration for safety · disadvantage

In the event that any adverse event develops to the subjects participating in the study, the attending physician promptly carries out the necessary response (examination, treatment, discontinuation of examination, etc.) and strives to ensure the safety of the subject.

Liver biopsy or hepatectomy involves various risks, but as shown in 3.1, liver biopsy or hepatectomy is required as part of daily practice irrespective of participation in this study It is targeted at the patient to be judged.

Among the tests added by participating in this study, blood tests and tests using ultrasound such as RTE, SWM, attenuation measurement, FibroScan, VTQ, SWE, SwSm, SWE_GE, or MR Touch, there is no disadvantage.

10.3. Independent Data Monitoring Committee and Kinki University Medical School Ethics Committee Approval

Regarding the implementation of this study, it is approved by the Independent Data Monitoring Committee and the Kinki University Medical School Ethics Committee and the Ehime University Graduate School of Medicine Ethics Committee.

11. Quality control and quality assurance.

11.1. Monitoring

11.1.1. Purpose

Confirm whether the test is performed safely and according to the test implementation plan and whether the data is correctly collected.

11.1.2. Central monitoring (in-house monitoring)

The data center conducts central monitoring with reference to the contents of the case report gathered by the research investigation management staff, RTE, liver biopsy results, with reference to the processing result of the digitized data. No facility visit monitoring is scheduled. Regular monitoring reports prepared by the data center will be submitted to the research representative and the independent data monitoring committee for consideration.

11.1.3. Contents

(1) Eligibility.

(2) Status of case accumulation, especially stop.

(3) Serious adverse events and their reporting status.

(4) Other.

12. Save record

The study responsible doctor keeps the following documents related to the implementation of the test etc. The storage period shall be until 5 years have passed since the publication of research results.

(1) Copy of Application Documents.

(2) Notification document from hospital director.

(3) Applications for various applications and reports of reports.

(4) Subject identification code list.

(5) Record on consent form, consent of subject.

(6) Basic data for preparing case reports (inspection data etc.).

13. Management of personal information and methods of anonymization

The pathological specimen and the ultrasonic examination data are managed by the case registration number given at the completion of registration. Specifically, at the time of case registration, personal identification information corresponding to the specimen is converted into alphanumeric characters, and thereafter, the specimen is treated only with the encoded concatenable anonymization number and used for analysis.

The case and sign reference table shall be kept strictly by the data center, and no clinical information that identifies the individual to the person in charge of the measuring institution will be transmitted.

14. Presentation of research results

- Register for clinical trial registration by the time of starting the study (www.clinicaltrials.gov) and submit it to the English journal after the final analysis is completed. Until the final analysis prescribed by the protocol, no announcement will be made except when approval by the independent data monitoring committee is obtained.
- However, the research representative or the research secretariat can publish the conference · paper (review) for the introduction of the research not including the analysis result of the research end point through the approval of the research representative and the Steering Committee .
- In principle, the author of the main publication of the research results shall be the head of the research secretariat, hereinafter the research representative, RTE, SWM, attendant of attenuation measurement data analysis person, independent pathologist interpreting committee member, statistical analysis director (for publication 1 person in charge at the time of analyzing), but it will decide in consultation with the research representative. Below that, according to the restriction by the contribution regulation of the paper, the faculty research officer is selected for each facility in order of the number of registrations, and it is made a coauthor.
- When writing several different papers, the leader consults with the Steering Committee and emphasizes facilities with a large number of registered cases.
- All coauthors should review the contents of the paper before submission and only those who agree on the content of the presentation. If agreement cannot be obtained in the discussion about contents, the researcher cannot include the researcher as coauthor after acknowledging Steering Committee.
- Because there is a possibility that the conference presentation may be repeated several times, we will announce it by circling from the research secretariat, the research representative, the researcher of the facility where there are many registrations or the doctor of the research execution facility. The presenter will decide the research representative with the approval of the representative group of the research organization. However, at the time of presenting the conference, the Research Secretariat is responsible for preparing the presentation and contents of presentation, and in principle, the Research Secretariat will contact the data center. Presenters other than the research secretariat cannot collect the results of aggregation / analysis directly from the data center without acknowledging the research secretariat and data center.

15. Clinical trial registration

This study is already scheduled to register in ClinicalTrial.gov (www.clinicaltrials.gov) or UMIN Clinical Trials Registry (UMIN - CTR).

16. Conflict of interest and research funding sources

We do not assume any conflict of interest that may affect the planning, implementation and presentation of this exam. Conflict of interest (conflict of interest) refers to interesting relationships that affect research outcomes, including money and personal relationships.

This study supplements research expenditure of GI internal medicine department of Kinki University School of Medicine and conducts research.

Decision making regarding planning, implementation and presentation of this exam is done by the research representative.

17. Response of expense of test participants and health damage

17.1. Expense of Study Participant

Of the tests adopted in this test, RTE, SWM, attenuation measurement, VTQ, SWE, SwSm, SWE_GE, and MR Touch are carried out at the burden of each testing facility because insurance treatment is not approved. Meanwhile, blood tests, liver biopsy or hepatectomy, FibroScan have been approved for medical insurance and are conducted within the scope of routine practice, so RTE, SWM, attenuation measurement, VTQ, SWE, SwSm, SWE_GE, Medical examination expenses including examination other than MR Touch, hospitalization expenses etc. are all paid by patient's insurance and self-payment.

17.2. Response of Health Damage

In the event of a health hazard in this test, appropriate treatment is carried out, and the cost is paid by the patient's insurance and self-pay as well as usual medical treatment. The doctor in charge prepares for liability before joining the liability insurance before the start of this examination.

18. Compliance with the test implementation plan, change

18.1. Termination, cancellation, suspension

18.1.1. Termination of the test

At all institutions, the time at which the follow-up from the end of the study implementation period (3 months from the last patient registration) is completed is set as the end of the study. At the end of the study at each facility, the investigator will promptly submit the study completion report to the hospital director and the research representative.

18.1.2. Cancellation of the test, interruption

The Independent Data Monitoring Committee reviews the validity of the continuation of the test as necessary. If it determines that continuation is inappropriate, the Committee recommends stopping or interrupting the study to this research organization. If this research organization decides to stop the examination in accordance with the recommendation, the research representative will inform the investigator as soon as possible as to discontinue, the reasons, and how to respond to the participant under examination. The investigator will report the circumstances to the Ethics Review Committee (or the IRB), and in accordance with the directions of the research representative and the Ethics Review Board (IRB), appropriate responses to participants under examination are taken.

The examination responsible doctor examines whether to continue the examination if it falls under the following matters.

1) When serious information on the safety and efficacy of test treatment is obtained.

2) When it is judged that it is difficult for recruitment of subjects to achieve planned case due to difficulty.

3) When the purpose of the test is achieved (by interim analysis etc.) before reaching the planned number of cases or scheduled test implementation period.

4) When the ethics review committee (IRB) has instructed to change the implementation plan etc. and it is judged that it is difficult to accept it.

The investigator will discontinue the examination if there is a recommendation or order to cancel by the Ethics Review Board (IRB). When deciding to stop or discontinue the study, the investigator will promptly report to the hospital director (or the head of each study facility) along with the reason.

18.2. Compliance with the test implementation plan

Researchers conducting this examination shall comply with this study plan unless the participants' safety and human rights are not impaired.

18.3. Deviation from the test implementation plan

1) The physician in charge shall not make any deviations or changes from the study plan before obtaining prior consent of the research representative and approval of the hospital director based on the prior review of the ethics review committee.

2) The physician in charge may deviate from the study plan or change it before obtaining prior consent of the research representative and prior approval of the ethics review committee for unavoidable reasons such as emergency avoidance it can. In doing so, the attending physician shall promptly submit the proposal to the Research Director and the Ethics Review Committee if the contents and reasons of deviation or change and revision of the test implementation plan etc. are necessary, and the research representative, The Ethics Review Committee and the hospital director.

3) In case of departure from the study plan, the attending physician records all deviant items along with the reasons, and the investigator considers the hospital director and the representative of the examination I have to report to. The investigator must preserve these copies.

18.4. Change of test plan

Changes to the test implementation plan and changes or revision of explanatory documents and agreements must be approved by the ethics review committee of each facility in advance. Also, if requested to change this Implementation Procedure in order to obtain approval, the investigator may change the test implementation plan at the facility after agreeing with the research representative.

18.4.1. Classification of changes in the test implementation plan

We handle changes in the test implementation plan after the approval of the Central Ethics Review Committee (Kinki University School of Medicine Ethics Committee) in two types, revision and revision. Definition and handling are as follows.

1) Amendment

Partial change in the test implementation plan that may increase the risk of the participants in the trial or related to the primary endpoint of the study.

It requires examination approval by the Independent Data Monitoring Committee and the Ethics Review Committee. If approval is received, indicate the approval date of the independent data monitoring committee on the cover page.

2) Revision

Modification of the test implementation plan which is unlikely to increase the risk of participants in the trial and is not related to the main evaluation items of the test.

Evaluation by the independent data monitoring committee is unnecessary, but it requires approval of the research representative and report to the independent data monitoring committee. Regarding approval of review by the Ethics Review Committee, follow the arrangements of each facility. In the case of approval, write the date of approval of the research representative on the cover page.

18.4.2 Approval of the Facility Ethics Committee at the time of revision / revision of the trial implementation plan

During the examination, with the approval of the Independent Data Monitoring Committee and amendments to this Study Implementation Plan or explanatory documents / agreement letters to the participants, the revised examination execution plan and explanatory documents are appointed by the Ethics Review Committee It must be approved by the Association (IRB).

Whether or not the change of contents is not a revision but a revision, the judgment of the ethics review committee will be required depending on the agreement of each facility.

If the revision is approved by the Ethics Review Committee, the investigator of each facility will send a copy of the Ethics Review Committee approval document to the data center. Documents approved by the Ethics Review Committee are kept by each facility and copies are kept by the data center.

19. Research organization

This study is a voluntary clinical trial carried out by research organizations. The relevant organization of this study will be shown below.

19.1. Research leader

Masatoshi Kudo (Department of Gastroenterology and Hepatology, Faculty of Medicine, Kindai University)

- Summary of clinical trials.
- Improvement of clinical trial implementation system.
- Determination of test implementation plan.
- Emergency response.

19.2. Research Bureau

Norihisa Yada

Department of Gastroenterology and Hepatology, Faculty of Medicine, Kindai University, Osaka, Japan, 589-8511.

TEL: +81-72-366-0221

E-mail: yada@med.kindai.ac.jp

19.3. Steering Committee (in no particular order, honorific title)

Masatoshi Kudo (Department of Gastroenterology and Hepatology, Faculty of Medicine, Kindai University)

Norihisa Yada (Department of Gastroenterology and Hepatology, Faculty of Medicine, Kindai University)

Shunji Sakurai (Department of Gastroenterology and Hepatology, Faculty of Medicine, Kindai University)

- Formulation of test plan, description document, consent form plan, examination of necessity of change, formulation of revision plan Presentation to research representative

19.4. Person in charge of RTE, SWM and attenuation measurement data analysis

Hitachi Aloka Medical, Inc. 2nd Medical System Engineering Division First Technology Development Department Development Section 2

3-1-1 Higashi Koigobuki, Kokubunji, Tokyo, 185-0014

TEL: +81-42-329-4519

E-mail: tono4143@hitachi-aloka.co.jp

19.5. Independent pathology interpretation committee (in no particular order, honorific title omitted)

Masayoshi Kage (Kurume University Pathology)

Michiie Sakamoto (Department of Pathology, Keio University Graduate School of Medicine)

Osamu Nakashima (Kurume University School of Medicine Pathology)

- Pathological analysis

19.6. Statistical analysis expert (honorific title omitted)

Kenichi Yoshimura (Kyoto University Medical School Hospital Discovery Medical Center)

- Statistical consideration on research design
- Determination of statistical analysis method
- Statistical advice on data aggregation / statistical analysis / publication

19.7. Independent Data Monitoring Committee (Effect Safety Evaluation Committee) (in no particular order, honorific title omitted)

Chairperson: Takashi Kumada (Ogaki City Hospital Gastroenterology Department)

Committee member: Masatoshi Tanaka (Yokokura Hospital Department of Gastroenterology)

Committee member: Hiroko Oka (Hosesaka Hospital)

- Monitoring for smooth and appropriate test implementation
- Implementation of intermediate analysis
- Review necessity of protocol revision based on implementation of interim analysis, validity of test continuation and recommendation to research representatives

19.8. Data center

Officer: Kimiko Yuge

TEL: +81-72 - 366 - 0221

E-mail: kin-live@med.kindai.ac.jp

20. Examination facility

The condition of the facility participating in this examination is that you are familiar with liver biopsy in the clinical department conducting the study and have HI VISION Ascendus by Hitachi Aloka Medical Co., Ltd., RTE, SWM and attenuation measurement can be performed.

20.1. Testing facility (honorific title omitted)

1. Department of Gastroenterology and Hepatology, Faculty of Medicine, Kindai University, Masatoshi Kudo.

2. Musashino Red Cross Hospital Gastroenterology and Hepatology, Namiki Izumi.

3. Departments of Gastroenterology and Metabology, Ehime University Graduate School of Medicine, Yoichi Hiasa.

1. References

1. Imbert-Bismut, F., et al., *Biochemical markers of liver fibrosis in patients with hepatitis C virus infection: a prospective study.* Lancet, 2001. **357**(9262): p. 1069-75.

2. Gressner, A.M., C.F. Gao, and O.A. Gressner, *Non-invasive biomarkers for monitoring the fibrogenic process in liver: a short survey.* World J Gastroenterol, 2009. **15**(20): p. 2433-40.

3. Castera, L., *Transient elastography and other noninvasive tests to assess hepatic fibrosis in patients with viral hepatitis.* J Viral Hepat, 2009. **16**(5): p. 300-14.

4. Castera, L., et al., *Prospective comparison of transient elastography, Fibrotest, APRI, and liver biopsy for the assessment of fibrosis in chronic hepatitis C.* Gastroenterology, 2005. **128**(2): p. 343-50.

5. Foucher, J., et al., *Diagnosis of cirrhosis by transient elastography (FibroScan): a prospective study.* Gut, 2006. **55**(3): p. 403-8.

6. Ganne-Carrie, N., et al., *Accuracy of liver stiffness measurement for the diagnosis of cirrhosis in patients with chronic liver diseases.* Hepatology, 2006. **44**(6): p. 1511-1517.

7. Sagir, A., et al., *Transient elastography is unreliable for detection of cirrhosis in patients with acute liver damage.* Hepatology, 2008. **47**(2): p. 592-5.

8. Tatsumi, C., et al., *Noninvasive evaluation of hepatic fibrosis using serum fibrotic markers, transient elastography (FibroScan) and real-time tissue elastography.* Intervirology, 2008. **51 Suppl 1**: p. 27-33.

9. Masuzaki, R., et al., *Risk assessment of hepatocellular carcinoma in chronic hepatitis C patients by transient elastography.* J Clin Gastroenterol, 2008. **42**(7): p. 839-43.

10. Millonig, G., et al., *Extrahepatic cholestasis increases liver stiffness (FibroScan) irrespective of fibrosis.* Hepatology, 2008. **48**(5): p. 1718-23.

11. Nguyen-Khac, E., et al., *The non-invasive diagnosis of cirrhosis using the Fibroscan must be performed with cause-specific stiffness cut-offs.* Gut, 2008. **57**(11): p. 1630; author reply 1630-1.

12. Lucidarme, D., et al., *Factors of accuracy of transient elastography (fibroscan) for the diagnosis of liver fibrosis in chronic hepatitis C.* Hepatology, 2009. **49**(4): p. 1083-9.

13. Wang, J.H., et al., *FibroScan and ultrasonography in the prediction of hepatic fibrosis in patients with chronic viral hepatitis.* J Gastroenterol, 2009. **44**(5): p. 439-46.

14. Itoh, A., et al., *Breast disease: clinical application of US elastography for diagnosis.* Radilogy, 2006. **239**:p.341-50.

15. Tsutsumi, M., et al., *Real-Time Balloon Inflation Elastography for Prostate Cancer Detection and Initial Evaluation of Clinicopathologic Analysis.* AJR, 2010. **194**(6):p.W471-6.

16. Fukunari, N., et al., *More Accurate and Sensitive Diagnosis for Thyroid Tumors with Elastography - Detection and Differential Diagnosis of Thyroid Cancers.*  MEDIX Supplement, 2007: p. 16-19.

17. Friedrich-Rust, M., et al., *Real-time elastography for noninvasive assessment of liver fibrosis in chronic viral hepatitis.* AJR Am J Roentgenol, 2007. **188**(3): p. 758-64.

18. Fujimoto, K., et al., *Non-invasive evaluation of Hepatic Fibrosis in patients with Chronic Hepatitis C using Elastography.* MEDIX Supplement, 2007: p. 24-27.

19. Friedrich-Rust, M., et al., *Real-time tissue elastography versus FibroScan for noninvasive assessment of liver fibrosis in chronic liver disease.* Ultraschall Med, 2009. **30**(5): p. 478-84.

20. Arena, U., et al., *Acute viral hepatitis increases liver stiffness values measured by transient elastography.* Hepatology, 2008. **47**:p.380-84.

21. Cobbold, J.F., et al., *Transient elastography in acute hepatitis: all that's stiff is not fibrosis.* Hepatology, 2008. **47**:p.370-72.

22. Colli, G., et al., *Decompensated chronic heart failure: increased liver stiffness measured by means of transient elastography.* Radiology, 2010. **257**:p.872-78.

23. Yada, N., et al., *Assessment of Liver Fibrosis with Real-time Tissue Elastography in Chronic Viral Hepatitis.* Oncology, 2013. **84 (suppl 1)**: p. 13-20.

24. Maklad, N.F., et al., *Attenuation of ultrasound in normal liver and diffuse liver disease in vivo.* Ultrason Imaging 1984;**6**:p.117-25.

25. Taylor, K.J., et al., *Quantitative US attenuation in normal liver and in patients with diffuse liver disease:Importance of fat.* Radiology 1986;**160**:p.65-71.

26. Wilson, L.S., et al., *Evaluation of ultrasonic attenuation in diffuse diseases of spleen and liver..* Ultrason Imaging 1987;**9**:p.236-47.

27. Garra, B.S., et al., *Quantitative estimation of liver attenuation and echogenicity: Normal state versus diffuse liver disease..* Radiology 1987;**162**:p.61-67.

28. Sasso, M., et al., *Controlled attenuation parameter(CAP): a novel VCTETM guided ultrasonic attenuation measurement for the evaluation of hepatic steatosis: preliminary study and validation in cohort of patients with chronic liver disease from various causes.* Ultrasound Med Biol. 2010;**36**(11):p.1825-35.
